# In Silico Identification of circPIM1/miR-16-5p/miR-195-5p/PIM1 Feed-Forward Loop in Recurrent Grade 2 Meningioma

**DOI:** 10.3390/ijms26178263

**Published:** 2025-08-26

**Authors:** Giuseppe Sotera, Carla Forte, Daniele Giuseppe D’Urso, Domenica Reina, Noemi Zuccaro, Andrea Giuseppe Toscano, Angela Caponnetto, Cristina Barbagallo, Giuseppe Broggi, Francesco Certo, Marco Ragusa, Rosario Caltabiano, Cinzia Di Pietro, Giuseppe Maria Vincenzo Barbagallo, Michele Purrello, Davide Barbagallo

**Affiliations:** 1 Department of Medical, Surgical Sciences and Advanced Technologies “G.F. Ingrassia”, Neurological Surgery, Policlinico Rodolico-San Marco University Hospital, University of Catania, Via Santa Sofia, 87, 95123 Catania, Italy; 2 Department of Biological, Geological and Environmental Sciences, University of Catania, Corso Italia, 57, 95129 Catania, Italy; 3 Department of Biomedical and Biotechnological Sciences, Section of Biology and Genetics “Giovanni Sichel”, University of Catania, Via Santa Sofia, 97, 95123 Catania, Italy; 4 Department of Biology and Biotechnology “Lazzaro Spallanzani”, University of Pavia, Via Adolfo Ferrata, 9, 27100 Pavia, Italy; 5 Department of Molecular Biotechnology and Health Sciences, Molecular Biotechnology Center “Guido Tarone”, University of Torino, Via Nizza, 52, 10126 Torino, Italy; 6 Department of Medical, Surgical Sciences and Advanced Technologies “G.F. Ingrassia”, Section of Anatomic Pathology, University of Catania, Via Santa Sofia, 87, 95123 Catania, Italy; 7 Interdisciplinary Research Centre on the Diagnosis and Therapy of Brain Tumors, University of Catania, Via Santa Sofia, 78, 95123 Catania, Italy; 8 Interdisciplinary Research Centre for the Study of Prevention, Diagnosis and Treatment of Tumors (PreDiCT), University of Catania, Via Santa Sofia, 97, 95123 Catania, Italy

**Keywords:** meningioma, recurrence, competitive endogenous RNA (ceRNA) network, feed-forward gene expression loop

## Abstract

In bulk meningioma (MNG) tumors, a biomarker based on the expression of 34 transcripts (34HR-MNG) has recently been described to be able to predict their outcome, including recurrence. To better study the molecular mechanisms regulating the expression of the 34HR-MNG transcripts and predict their functional involvement in MNG recurrence, we built a competitive endogenous RNA (ceRNA) network through an in silico approach. MiRNAs targeting 34HR-MNG transcripts and corresponding sponging circRNAs were retrieved through MiRTarbase and ENCORI databases, respectively. The expression of candidate circRNA host genes belonging to the 34HR-MNG transcripts was correlated with specific molecular and clinical features of 89 and 20 WHO grade 1 and 2 MNGs, respectively, by querying the RNA-seq dataset GSE189672. The expression of candidate circRNAs and their host gene was validated through qRT-PCR. Among the 34HR-MNG transcripts, the Pim-1 proto-oncogene, serine/threonine kinase (PIM1) was significantly upregulated in (i) WHO grade 2 vs. grade 1 and (ii) recurrent vs. not recurrent WHO grade 2 MNGs. PIM1 expression positively and negatively correlated with that of Ki-67 and NF2, respectively, in recurrent WHO grade 2 MNGs. CircRNAs 0076215 and 0076216, both generated from the PIM1 host gene, were predicted to sponge miRNAs 16-5p and 195-5p, two tumor suppressors in MNG, in turn targeting PIM1. The expression of circRNAs 0076215 and 0076216, validated for the first time in a set of 19 physiological human tissues, positively correlated with that of their host gene (Rho value = 0.579 and 0.681, *p*-value = 0.026 and 0.013, respectively). Our data suggest that PIM1 is an oncogene involved in the recurrence of WHO grade 2 MNG and that the upstream ceRNA network, comprising circRNAs 0076215 and 0076216 and miRNAs 16-5p and 195-5p, is responsible for its upregulation through a feed-forward loop.

## 1. Introduction

Meningiomas (MNGs) are the most common primary central nervous system (CNS) tumors, mainly arising from the meninges [1]. In 2021, the World Health Organization (WHO) introduced new molecular criteria for the categorization of MNGs, adding new features to the classical classification of three grades, in line with other CNS tumors [2]. Driver mutations, involving genes such as NF2, moesin-ezrin-radixin-like (MERLIN) tumor suppressor (NF2), SWI/SNF-related BAF chromatin remodeling complex subunits B1 and E1 (SMARCB1, SMARCE1), TNF Receptor-Associated Factor 7 (TRAF7), Kruppel-like Factor 4 (KLF4), different genes belonging to the phosphoinositide 3-kinase (PI3K) and Hedgehog (HH) signaling pathways, and RNA Polymerase II Subunit A (POLR2A), in conjunction with other exogenous factors, are known to influence the onset, recurrence, grade, location, and other characteristics of MNGs [3].

MicroRNAs (miRNAs) are a class of small non-coding RNAs that play a crucial role in the regulation of gene expression at the post-transcriptional level by binding to complementary sites on target mRNAs [4]. These tiny regulatory molecules may be used as risk or protective biomarkers in cancer and represent new druggable targets [5,6]. Several miRNAs are involved in MNG pathogenesis and recurrence: the downregulation of miR-331-3p in conjunction with partial tumor resection are predictive factors for MNG recurrence [7]; miR-200a is downregulated in recurrent MNGs when compared with not recurrent ones and it has also been suggested as a promising biomarker for liquid biopsies [8]; miR-224-5p is upregulated in MNG with respect to healthy control tissue and acts as a suppressor of the ERG2-BAK-mediated apoptotic pathway [9]; and miR-16 and miR-519 are downregulated in MNG when compared with physiological arachnoid cells and their dysregulation contributes to cell growth in concert with the RNA-binding protein HuR [10].

Circular RNAs (circRNAs), single-stranded RNAs with a covalently closed-loop structure, have been described as critical regulators of gene expression [11,12]. By entering into the so-called competitive endogenous RNA (ceRNA) networks, circRNAs compete with mRNAs for the binding of miRNAs [13,14]. CircRNAs are often aberrantly expressed in cancer, where they can exert oncogenic or tumor-suppressive functions, depending on their interactants and cell context [15,16]. Because of their abundance, stability, and tissue- and developmental-specific expression patterns, circRNAs may be used as biomarkers and therapeutic targets [15]. To the best of our knowledge, only one circRNA (hsa_circ_0004872) has been described to date as being involved in MNG pathogenesis by acting as a tumor suppressor, thanks to its ability to tether and suppress the onco-miR 190a-3p and to compete with Programmed death-ligand 1 (PD-L1) mRNA for the binding of the RNA-binding protein (RBP) eukaryotic translation initiation factor 4A3 (EIF4A3), determining a significant decrease in PD-L1 protein and the consequent repression of MNG cells [17,18]. Apart from hsa_circ_0004872, the study of ceRNA networks in MNG has been limited to some long non-coding RNAs (lncRNAs), such as the small nucleolar RNA host gene 1 (SNHG1) [19], metastasis-associated lung adenocarcinoma transcript 1 (MALAT1) [20], LINC00702 [21], invasive meningioma-associated transcript 1 (IMAT1) [22], LINC00460 [23], and maternally expressed gene 3 (MEG3) [24].

Although most MNGs are classified as benign (WHO grade 1), their intrinsic biological features, together with their anatomical location (which may pose surgical challenges), can significantly influence symptoms, treatment strategies, recurrence, and patient outcome, independent of their grade [25,26,27]. Knowledge of MNG biology has increased dramatically over the last few years, leading to a deeper stratification of this tumor, thanks to the growing collection of genomic, transcriptomic, and epigenomic data [2,28]. More specifically, the integration of *omics* data allows researchers to distinguish between subclasses of MNG showing different risks in terms of recurrence, response to radiotherapy, and outcome [29,30,31,32,33]. Among MNGs, WHO grade 2 has an average 5-year recurrence rate of between 40% and 50% after gross total resection [34,35], representing an intermediate group of MNGs between the more favorable and unfavorable prognoses of WHO grades 1 and 3 MNGs, respectively. For this reason, studying ceRNA networks involved in grade 2 MNG recurrence may contribute to their subtypization into low-risk (LR) and high-risk (HR) groups and may provide new information on the molecular mechanisms underlying the recurrence of grade 2 MNGs, supplying new potential druggable targets for the treatment of HR patients.

Recently, Chen and colleagues reported a biomarker made of 34 transcripts (henceforth 34HR-MNG) whose expression in bulk tumors after surgery and before chemotherapy or radiotherapy can predict the outcome (comprised recurrence) of MNGs, independent of their grade or other histological features, and outperforming previously used prognostic biomarkers [36].

In this scenario, with the aim of expanding our knowledge of the molecular mechanisms regulating the expression of the 34HR-MNG through an in silico approach, we built a ceRNA network potentially involved in the recurrence of WHO grade 2 MNG, specifically focusing on PIM1 (one of the 34HR-MNG transcripts) and its upstream miRNA and circRNA regulators.

## 2. Results

### 2.1. Identification of Candidate miRNAs Regulating the Expression of 34HR-MNG Transcripts

According to miRTarBase, 32 miRNAs (henceforth candidate MNG recurrence (MR)-miRNAs) target 24 out of 34HR-MNG transcripts (Table S1). MiR-124-3p shows the highest number (10) of targets among the 34HR-MNG (Table S1). The most represented family is miR-15, with a total of 6 members out of 8 annotated in MirGeneDB 2.1 (Table S1). Functional annotation through MiRPath 4.0 revealed that pathways involved in cancer and cell cycle control are among the top 10 significantly enriched, confirming the potential involvement of candidate MR-miRNAs in MNG recurrence [37] (Table S2). Cell cycle and cell division are consistently among the top 10 biological processes regulated by MR-miRNAs (Table S3).

### 2.2. Identification of circRNAs Sponging Candidate MR-miRNAs

Analysis through ENCORI identified 96 candidate circRNAs synthesized by 11 host genes (CCND2; CDK6; CHEK1; CKS2; COL1A1; EZH2; FBLIM1; FGFR4; MDM4; MYBL1; and PIM1) that were (i) belonging to 34HR-MNG and (ii) predicted targets of MR-miRNAs (Table S4). Among the candidate circRNAs, four of them (hsa_circ_0010090, hsa_circ_0044516, hsa_circ_0044520, and hsa_circ_0044529), deriving from FBLIM1 and COL1A1 host genes, are known to be upregulated in hepatocellular carcinoma, prostate cancer, gastric cancer, lung cancer, and laryngeal squamous cell carcinoma, according to the circRNADisease v2.0 database (Table S5); there was no further information for the other circRNAs, according to the same database. The biological function and any known involvement in the pathophysiology of MNG, particularly recurrence, of the 11 candidate host genes are reported in Table S6: apart from CCND2 and CDK6, whose suppression within 34HR-MNG contrasts with their known oncogenic biological function, the altered expression of the remaining 9 host genes is in line with their documented oncogenic or tumor-suppressive functions. According to ENCORI, the 96 candidate circRNAs (henceforth candidate MR-circRNAs) show a median of five binding sites (MBS) for the same or different candidate MR-miRNAs within their sequences. Fifty-one MR-circRNAs, biogenerated from seven host genes (CCND2, CDK6, CHEK1, COL1A1, FGFR4, MDM4, and PIM1), have a number of MBS greater than or equal to the median value of five (Table S7). In general, and as expected, we observed that the higher the size of MR-circRNA, the higher the number of MBS revealed (Rho value = 0.62, *p*-value = 0, Spearman’s correlation test). COL1A1, MDM4, FBLIM1, CCND2, PIM1, and CHEK1 are the host linear transcripts from which most MR-circRNAs are generated, with 60, 8, 8, 6, 4, and 3 different MR-circRNAs generated, respectively (Table S8).

### 2.3. PIM1 Is Upregulated in Recurrent WHO Grade 2 MNGs

**Figure 1:**
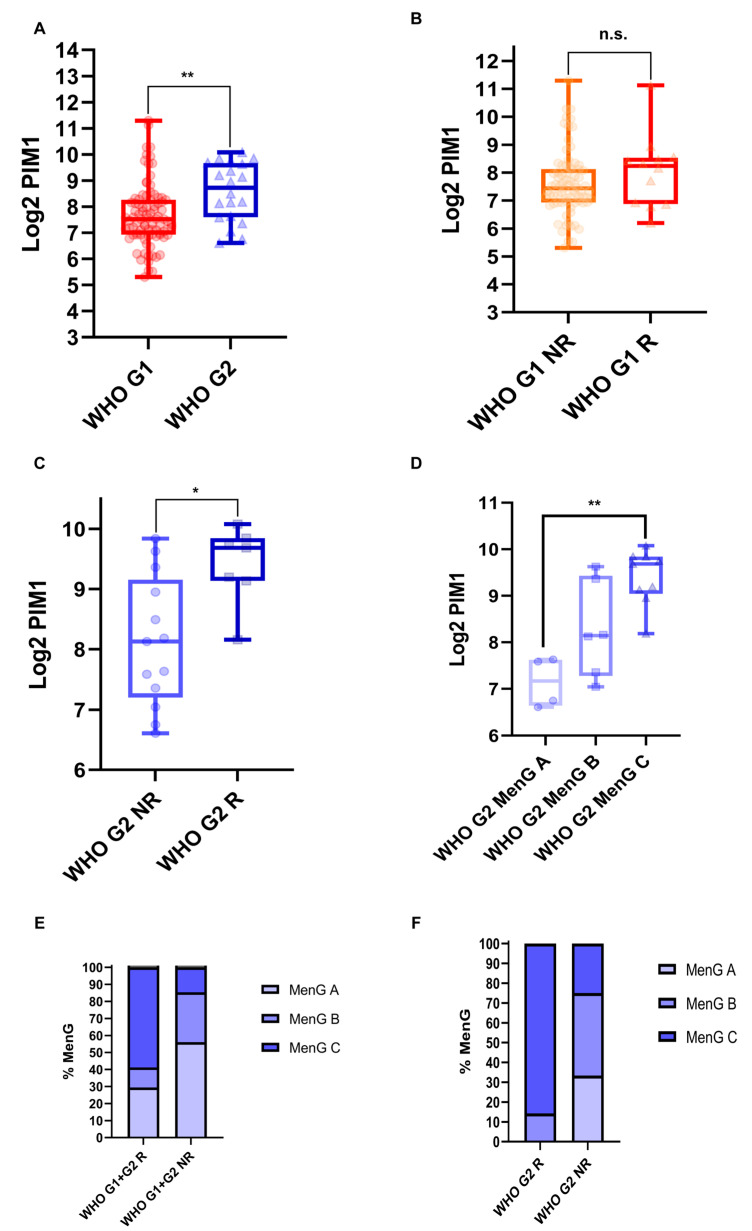
PIM1 expression in MNG. (**A**) Expression of PIM1 in WHO grade 1 (G1) and 2 (G2) MNGs; (**B**,**C**) expression of PIM1 in recurrent (R) and not recurrent (NR) G1 and G2 MNGs; and (**D**) expression of PIM1 in MenG A, B, and C G2 MNG subtypes. Data are represented as box plots of Log2 of DESeq2_vst normalized data of PIM1; n.s. = not significant; * = *p*-value < 0.05; ** = *p*-value < 0.01. (**E**,**F**) Stacked bar plots representing the prevalence (as %) of MenG subtypes in the entire subtyped cohort of R and NR G1 plus G2 (**E**) and G2 only (**F**) MNGs.

Prediction of MR-miRNAs and MR-circRNAs involved in the regulation of 34HR-MNG identified CHEK1 and PIM1 as the unique transcripts entering into a feed-forward loop ceRNA network: CHEK1 and PIM1 are the validated targets of four miRNAs (miR-16-5p, miR-124-3p, miR-193b-3p, and miR-195-5p), described as tumor suppressors in MNG or other cancers in the literature, which, in turn, may be sponged by MR-circRNAs biogenerated by the same CHEK1 and PIM1 host genes (Table 1). Interactions among MR-miRNAs, circCHEK1, and circPIM1 are corroborated by significant AGO-CLIP region *p*-values (Table 1). Based on the GSE189672 dataset, among candidate MR-circRNAs’ host genes, PIM1 is significantly upregulated in (i) WHO grade 2 vs. grade 1 MNGs (fold-change (FC) between median values = 2.3, *p*-value = 0.0021, unpaired Mann–Whitney test) (Figure 1A) and (ii) recurrent vs. not recurrent WHO grade 2 MNGs (FC between median values = 2.95, *p*-value = 0.011, unpaired Mann–Whitney test) (Figure 1C). No significant differential expression was retrieved between recurrent and not recurrent WHO grade 1 MNGs (*p*-value = 0.22, unpaired Mann–Whitney test) (Figure 1B). PIM1 is also significantly upregulated in MenG C (the most aggressive MNG subtype, based on the MNG classification proposed by Bayley et al. in 2022 [33]) vs. MenG A WHO grade 2 MNGs (FC = 1.96, *p*-value = 0.0051, Kruskal–Wallis test corrected with Dunn’s post-hoc test) (Figure 1D). In the same GSE189672 dataset, and according to the literature, MenG C is the most prevalent subtype in recurrent when compared with not recurrent MNGs, across the three MenG subtypes (with a frequency of 58.8% within 17 recurrent MNGs against 14.6% within 82 not recurrent MNGs, *p*-value = 0.0012 and 0.0059, Fisher exact test for matches MenG C vs. MenG A and MenG C vs. MenG B, respectively) when all the subtyped MNGs (*n* = 99 on 109) are considered (Figure 1E). The association between the MenG C MNG subtype and its ability to recur is also confirmed when only WHO grade 2 MNGs (*n* = 19 subtyped on 20) are analyzed (Figure 1F): a higher percentage (85.7%) of recurring WHO grade 2 MNGs were subtyped as MenG C, against 14.2% of MenG B and 0% of MenG A. The distribution of the three MenG subtypes appears more equal (33.3, 41.7, and 25% for MenG A, B, and C, respectively) in not recurring WHO grade 2 MNGs (Figure 1F).

### 2.4. PIM1 Upregulation Is Associated with the Most Aggressive Molecular MNG Phenotype

**Figure 2:**
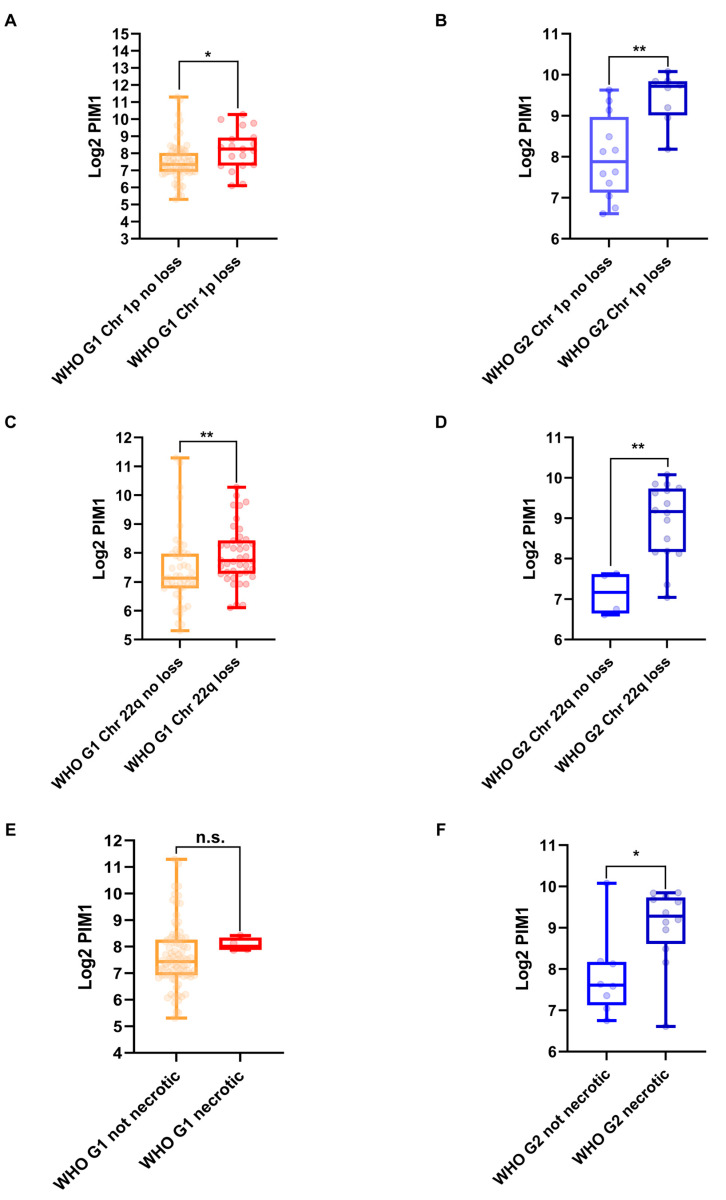
PIM1 expression in different molecular and histological MNG subtypes. Expression of PIM1 in WHO G1 (**A**) and G2 (**B**) MNGs with and without chromosome 1p (chr 1p) loss; WHO G1 (**C**) and G2 (**D**) MNGs with and without chromosome 22q (chr 22q) loss; WHO G1 (**E**) and G2 (**F**) MNGs without and with a necrotic histological feature. Data are represented as box plots of Log2 of DESeq2_vst normalized data of PIM1; n.s. = not significant; * = *p*-value < 0.05; ** = *p*-value < 0.01.

**Figure 3:**
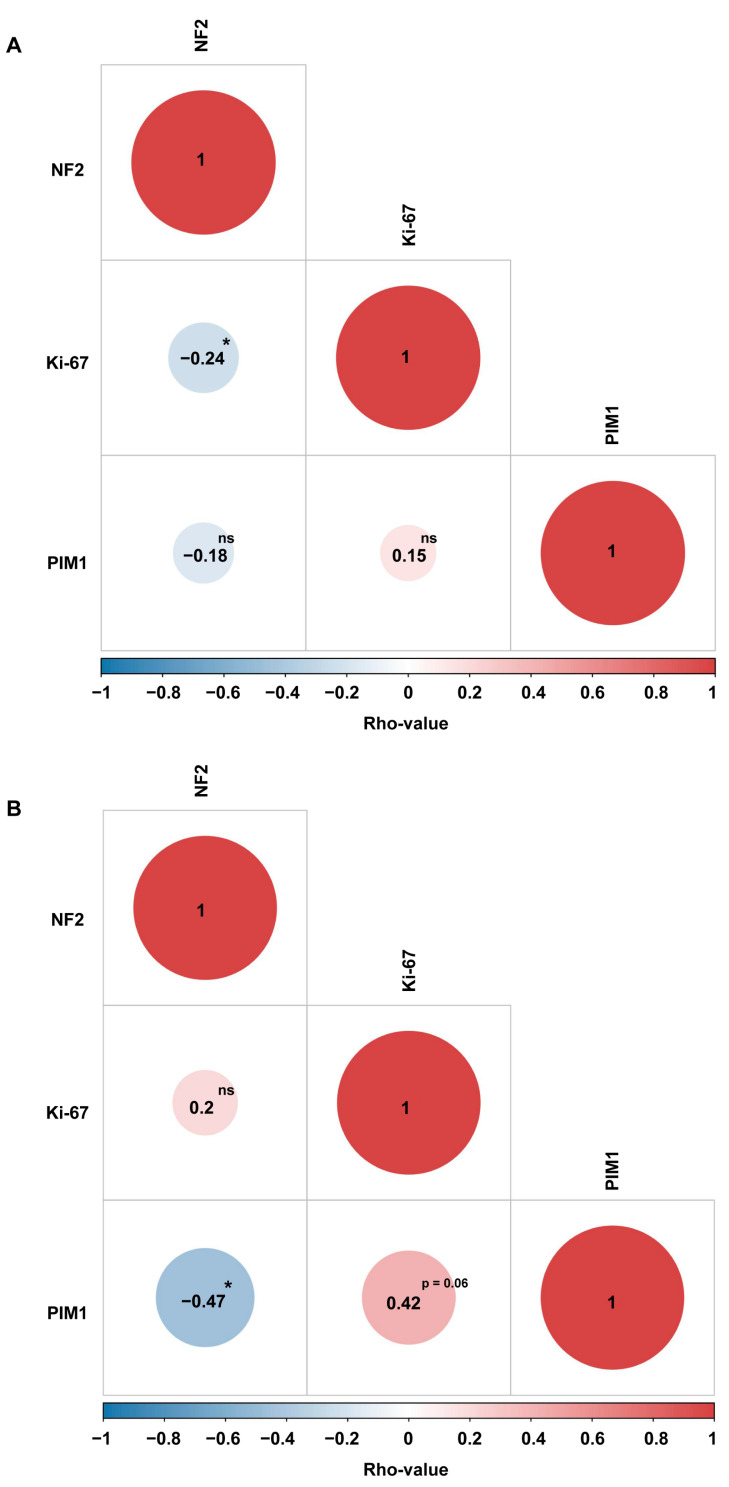
Correlogram showing correlations among PIM1 expression, NF2 expression, and Ki-67 index in WHO grade 1 (**A**) and 2 (**B**) MNGs. The color of the circle is linked to the correlation type (colors from light red to dark red and from light blue to dark blue are representative of positive and negative correlation, respectively); the size of the circle is proportional to the strength of correlation (the higher the absolute Rho-value, the larger the size of the circle). Corrplot v. 0.1.0 (https://hiplot.cn/basic/corrplot, accessed on 1 July 2025) was used to generate the chart. (* *p*-value < 0.05, ns = not significant).

Based on data retrieved from the GSE189672 dataset, PIM1 is significantly upregulated in WHO grade 1 and 2 MNGs, showing a loss of chromosomes 1p and 22q. In both cases, the highest FC is observed in WHO grade 2 MNGs (FC = 3.57 and 3.99, *p*-value = 0.0015 and 0.005, respectively, unpaired Mann–Whitney test) (Figure 2A–D). Necrotic WHO grade 2 MNGs also show a significantly higher expression of PIM1 when compared with non-necrotic MNGs of the same grade (FC = 3.18, *p*-value = 0.03, unpaired Mann–Whitney test) (Figure 2F). No significant differential expression of PIM1 is observed between necrotic and non-necrotic WHO grade 1 MNGs (Figure 2E). PIM1 shows a significant negative and positive correlation with NF2 and Ki-67 expression, respectively (Rho-value = −0.47 and 0.42, *p*-value = 0.037 and 0.06, Spearman’s correlation test, respectively), specifically in WHO grade 2 MNGs (Figure 3).

### 2.5. MR-CircRNAs 0076215 and 0076216 Positively Correlates with PIM1

**Figure 4:**
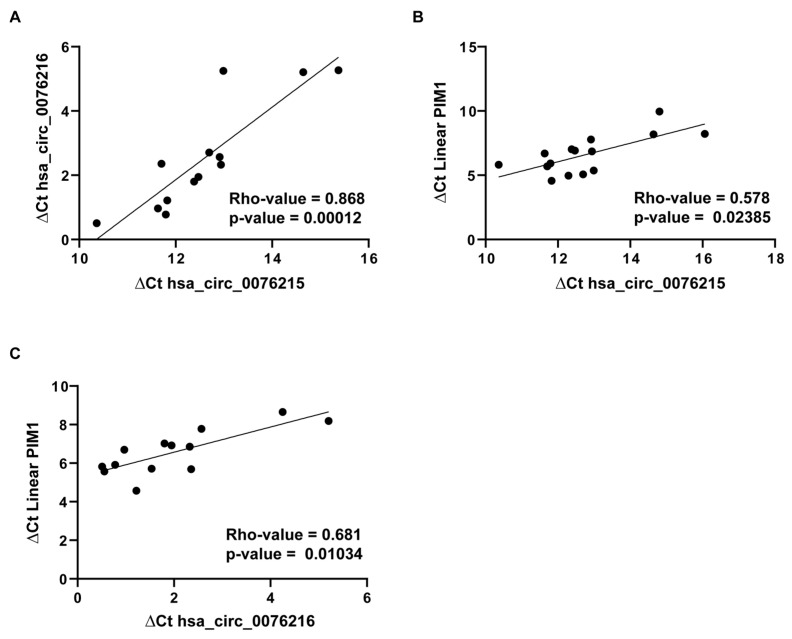
Correlation among MR-host gene PIM1 and MR-circRNAs 0076215 and 0076216 expression. Scatter plots showing correlation between (**A**) MR-circRNAs 0076215 and 0076216; (**B**) MR-host gene PIM1 and MR-circRNA 0076215; and (**C**) MR-host gene PIM1 and MR-circRNA 0076216 in a panel of 19 human physiological tissues. Expression data are reported as ∆Cts, obtained by using GAPDH as a reference transcript. Spearman’s correlation test was applied to retrieve the correlation. Rho and *p*-values are reported within each panel of the figure.

The expression of MR-circRNAs 0076215 and 0076216 and their host gene PIM1 was validated in a panel of 19 commercially available human physiological tissues. An analysis of correlation highlighted a significant positive correlation between circRNAs 0076215 and 0076216 (Rho-value = 0.87, *p*-value = 0.0001, Spearman’s correlation test) and their host gene PIM1 (Rho-value = 0.58 and 0.68, *p*-value = 0.02 and 0.01, Spearman’s correlation test, respectively) (Figure 4A–C). In silico analysis of KEGG pathways, performed through the GeneSet analysis tool in R2 genomics (https://hgserver1.amc.nl/cgi-bin/r2/main.cgi, accessed on 1 November 2024) within the GSE189672 dataset, showed that genes positively correlated with PIM1 are enriched in cancer-related pathways (Figure S1).

### 2.6. MR-miRNAs and PIM1 Are Expressed in Meningeal Tissue

The expression of candidate MR-miRNAs and PIM1 was checked at the tissue and single-cell level. Candidate MR-miRNAs 16-5p and 195-5p are expressed in the brain, dura mater, arachnoid mater, bone, and vein, although at different levels (Figure S2). By querying the GSE150219 dataset, we found that Pim1 was mainly expressed in proliferating fibroblasts of embryonal murine meninges (Figure S3). The ability of MR-circRNAs 0076215 and 0076216 to bind hsa-miR-16-5p and hsa-miR-195-5 was strongly supported by AGO-CLIP-seq data from ENCORI and by the CircInteractome tool. The latter identified AGO1 and AGO2, together with HuR and MOV10, as RBPs showing a significantly higher number of binding sites within MR-circRNAs 0076215 and 0076216 sequences when compared with 139 randomly chosen circRNAs of a similar size (Multiple Mann–Whitney test, False Discovery Rate set at 10%) (Figure S4). The expression verified at the tissue and single-cell level, and the correlations retrieved through qRT-PCR corroborated the reliability of the suggested MR-ceRNA network involving MR-circRNAs 0076215, 0076216, MR-miRNAs 16-5p, 195-5, and PIM1 in the meninges and its potential dysregulation in recurrent WHO grade 2 MNG.

## 3. Discussion

In recent years, our knowledge concerning MNG biology has significantly improved. Above all, the epigenetic pathways involved in MNG recurrence have been under investigation, and their association with known genetic and morphological features of the tumor is leading to a re-classification of MNG with important results in terms of the discovery of new diagnostic, prognostic, and therapeutic targets [2]. In this scenario, understanding how gene expression regulatory networks work in MNG cells is critical. Through this study, we aimed to increase our knowledge in this field by investigating ceRNA networks, involving circRNAs, miRNAs, and their targets, potentially regulating the expression of 34 transcripts known to be dysregulated in HR-MNGs. As far as we know, only two manuscripts have, to date, focused on a unique candidate circRNA (hsa_circ_0004872) that acts as a sponge for miR-190a-3p, by using a targeted, low-throughput approach [17,18]. Our stringent and funnel-like in silico and wet lab analyses allowed us to focus on two MR-circRNAs (hsa_circ_0076215 and hsa_circ_0076216), synthesized by the common host gene PIM1 and, in turn, predicted to sponge two tumor suppressor miRNAs (miR-16-5p and miR-195-5p) showing PIM1 among their validated targets. PIM1 is a known oncogene, upregulated in GBM [52] and in several other cancers where it contributes to recurrence and drug resistance [53]; however, its specific involvement in MNG is, to date, unknown. Furthermore, thanks to its role in the regulation of the cell cycle, PIM1 is a critical druggable target in cancer, and interest in this field is increasing, as suggested by several drugs that have been developed against it [54]. By querying the GSE189672 dataset, we propose PIM as an oncogene in MNG. In line with the latest Central Brain Tumor Registry of the United States (CBTRUS) statistics [1], the GSE189672 dataset consists of about 82% (89 samples out of 109) of WHO grade 1 and the remaining 18% (20 samples out of 109) of WHO grade 2 MNGs. The same distribution was observed between recurrent and not recurrent MNGs within the GSE189672 dataset. PIM1′s upregulation in WHO grade 2 vs. grade 1 and in recurrent grade 2 vs. not recurrent grade 2 MNG, in necrotic vs. non-necrotic grade 2 MNGs, and in chromosome 1p and 22q-loss MNGs, as well as positive and negative correlation between its expression and Ki-67 and NF2 expression, respectively, suggest its oncogenic function in MNG. Necrotic and chromosomes 1p/22q-loss harboring MNGs show an aggressive phenotype and a higher probability to recur [33,55]. Up- and downregulation of Ki-67 and NF2, respectively, is also linked with recurring MNGs [56,57]. PIM1 overexpression in recurrent MNGs may resemble murine meninges development, where its orthologue Pim1 is quite exclusively expressed in proliferating meningeal fibroblasts, based on the GSE150219 dataset [58]. Our data strongly suggest that PIM1 expression may be regulated through a feed-forward gene expression loop involving MR-miRNAs 16-5p and 195-5p and their sponges MR-circRNAs 0076215 and 0076216. The crosstalk between circRNAs and their linear host transcripts, as well as several mechanisms of feed-forward gene expression loops mediated by ceRNAs in physiological and pathological conditions, have been reported in the literature [59,60,61,62,63,64,65]. The ceRNA network engaging MR-circRNAs 0076215 and 0076216 and PIM1 is strengthened by the positive correlation observed among these molecules in several physiological cells, tissues, and organs, including astrocytes and the brain as representatives of CNS cellular components. To the best of our knowledge, the expression of hsa_circ_0076215 and hsa_circ_0076216 was experimentally validated by real-time PCR for the first time in this study, and no data about their function have been reported in the literature to date. More information is available on candidate MR-miRNAs and PIM1, although no data reporting the link between the latter and MNG are present in the literature to date. Several studies show that miR-16-5p is dysregulated in different tumors [66], and it is also a known downregulated tumor suppressor in CNS cancers such as glioblastoma (GBM) [67] and gliomas [68], further to MNG itself, where it is downregulated [10]. Due to these features, miR-16-5p constitutes an attractive therapeutic target [69,70] and a potential biomarker for cancer diagnosis [71,72]. PIM1 is a validated target of miR-16-5p [68,73]. MiR-195-5p is downregulated in higher grade MNGs and several of its targets are upregulated and linked to dysregulated signaling pathways in higher grade or aggressive MNGs [74]. MiR-195-5p, potentially sponged by five candidate MR-circRNAs, acts as a tumor suppressor in malignant MNG, by directly targeting the upregulated fatty acid synthase (FASN), whose role in de novo lipogenesis is critical for both the proliferation and survival of tumor cells [75]. Interestingly, FASN is positively correlated to PIM1 expression in MNG, based on the GSE189672 dataset. Furthermore, the vascular endothelial growth factor A (VEGFA) is another validated target of miR-195-5p, and its upregulation correlates with higher grade MNGs and a higher probability of MNG recurrence [76,77]. PIM1 is also a validated target of miR-195-5p [78,79].

## 4. Materials and Methods

### 4.1. Identification of Candidate miRNAs Involved in MNG Recurrence

MiRNAs validated to regulate the expression of 34HR-MNG transcripts [28] were retrieved by querying the miRTarBase database (https://mirtarbase.cuhk.edu.cn/~miRTarBase/miRTarBase_2025/php/index.php, accessed on 1 August 2023) [80]. MiRNAs having at least four different targets, among 34HR-MNG, were used for downstream analysis. Connection to specific miRNA families was queried through MirGeneDB 2.1, accessed on December 2024 [81]. MiRPath 4.0 (http://62.217.122.229:3838/app/miRPathv4, accessed on 1 August 2024) [82] was used to functionally annotate candidate miRNAs: as input parameters, TarBase v8.0 was used for the selection of validated targets; miRbase v22.1 was used for the selection of miRNA annotation; and KEGG pathways and GO terms were chosen to perform the pathways and biological process enrichment analysis, respectively.

### 4.2. Identification of Candidate circRNAs Involved in MNG Recurrence

The ENCORI (Encyclopedia of RNA Interactomes) database (https://rnasysu.com/encori/, accessed on 1 August 2024) [83], a database that stores AGO CLIP-seq data from thousands of datasets (accessed on 1 January 2024), was queried to predict circRNAs sponging candidate miRNAs. Specific candidate circRNA isoforms were identified through ENCORI by selecting each single interaction with candidate miRNAs. The circRNADisease v2.0 database [84] (accessed on 1 August 2024), was queried to check for known involvement of candidate circRNAs in disease. The CircInteractome tool [85] (https://circinteractome.irp.nia.nih.gov/, accessed on 1 May 2025) was queried to annotate the number of predicted RBP binding sites within circRNA sequences.

### 4.3. Correlation Between MR- circRNA Host Gene Expression and MNG Clinical and Molecular Data

RNA-seq data from a cohort of 109 MNGs reported by Bayley and colleagues (GSE189672) [33] were retrieved and analyzed through the “R2: Genomics Analysis and Visualization Platform” https://hgserver1.amc.nl/cgi-bin/r2/main.cgi, accessed on 1 November 2024. The dataset consists of 89 and 20 WHO grade 1 and 2 MNGs, respectively, according to the 2016 WHO classification, which did not receive any radiotherapy or chemotherapy before sampling. More data on the inclusion and exclusion criteria of the cohort analyzed are reported in the Supplementary Material.

Gene expression data of candidate host genes were correlated with outcome (recurrence); methylation subtypes; age at surgery; sex; WHO grade (1/2); necrosis; Simpson grade; Ki-67 index; recurrence-free time; large-scale chromosome deletions; loss of chromosomes 22q and 1p; TERT promoter mutation; NF2 expression; and NF2, TRAF7, PIK3CA, AKT1, ARID1A, SMO, and KLF4 mutations. Only statistically significant correlations (*p*-value < 0.05) have been reported in the results section.

### 4.4. Tissue and Single-Cell Expression Analysis

Expression of candidate MR-miRNAs within the brain, dura mater, arachnoid mater, bone, and vein was ascertained by the miRNA TissueAtlas 2025 database (https://ccb-web.cs.uni-saarland.de/tissueatlas2/tissues, accessed on 1 December 2024) [86]. PIM1 expression was assessed through single-cell RNA-seq (scRNA-seq) data within the dataset of Meningeal Fibroblast scRNA-seq by DeSisto and colleagues (GSE150219) [58]. The platform https://cuanschutz-devbio.shinyapps.io/Siegenthaler_shiny/ (accessed on 1 December 2024) was queried to map the expression of Pim1 within meningeal fibroblast cells during mouse embryo development.

### 4.5. qRT-PCR and ceRNA Network

The expression of candidate MR-circRNAs and the PIM1 host gene was assayed through one-step qRT-PCR on a panel of 19 commercially available physiological human tissues (spleen, adipose tissue, kidney, skeletal muscle, cervix, prostate, trachea, colon, ovary, lung, small intestine, astrocytes, thymus, placenta, testis, heart, esophagus, bladder, and brain) (FirstChoice^®^ Human Total RNA Survey Panel, ThermoFisher Scientific, Waltham, MA, USA). PCR reactions were performed through a QuantStudio™ 7 Flex Real-Time PCR System (ThermoFisher Scientific, Waltham, MA, USA) by using the Power SYBR™ Green RNA-to-CT™ 1-Step Kit (ThermoFisher Scientific, Waltham, MA, USA). Raw Ct values were exported through the QuantStudio™ Real-Time PCR software v1.3. GAPDH was used as the reference transcript to calculate ∆CT values. The primer sequences used in this study are reported in Table S9.

### 4.6. Statistics

All the statistical tests performed in this study are reported throughout the text. The Shapiro–Wilk test was performed upstream to check for normal distribution of PIM1 expression data in the whole cohort. Due to the non-normal distribution of PIM1 expression, non-parametric statistical tests were used for data analysis. The *p*-values and corrected *p*-values < 0.05 were considered significant.

## 5. Conclusions

This study suggests for the first time that PIM1 is a new candidate oncogene promoting WHO grade 2 MNG recurrence. Our data also suggest that PIM1 expression may be regulated at the post-transcriptional level by a ceRNA network involving miRNAs 16-5p and 195-5p and the circRNAs hsa_circ_0076215 and hsa_circ_0076216, through a feed-forward gene expression loop. Although this is mainly an in silico study and data need to be validated experimentally to include cohorts of MNG samples at several grades of malignancy, comprising WHO grade 3, prospectively, it paves the way to the identification of ceRNA networks involved in MNG progression and recurrence, and stimulates the idea that the investigation of gene regulatory networks should be added to gene expression biomarkers to strengthen their power of prediction.

## Figures and Tables

**Table 1:** Identification of MR-ceRNA networks. For each MR-miRNA described as a tumor suppressor in MNG or other cancers in the literature (see reference column), the corresponding MR-circRNAs sponging it, the MR-host linear transcript targets, and the AGO-CLIP region *p*-value, are reported. CircBase IDs identify each candidate MR-circRNA biogenerated either by CHEK1 or PIM1.

MR-miRNA	Function in MNG or Other Cancers	References	Candidate MR Host Linear Transcripts	Candidate MR-circRNAs (Biogenerated by MR Host Linear Transcripts and Acting as a Sponge for MR-miRNAs)	AGO-CLIP Region *p*-Value
hsa-miR-16-5p	Tumor suppressor in MNG	[10]	CHEK1	circCHEK1 (hsa_circ_0024791; hsa_circ_0024793; hsa_circ_0024794)	≤10^−6^
hsa-miR-16-5p	Tumor suppressor in MNG	[10]	PIM1	circPIM1 (hsa_circ_0076213; hsa_circ_0076214; hsa_circ_0076215; hsa_circ_0076216)	≤10^−12^
hsa-miR-124-3p	Tumor suppressor in several cancers	[38,39,40,41,42,43,44,45,46,47,48,49] *	PIM1	circPIM1 (hsa_circ_0076213; hsa_circ_0076214)	≤10^−12^
hsa-miR-193b-3p	Tumor suppressor in MNG	[50]	CHEK1	circCHEK1 (hsa_circ_0024793)	≤10^−8^
hsa-miR-195-5p	Tumor suppressor in MNG	[51]	CHEK1	circCHEK1 (hsa_circ_0024793)	≤10^−6^
hsa-miR-195-5p	Tumor suppressor in MNG	[51]	PIM1	circPIM1 (hsa_circ_0076213; hsa_circ_0076214; hsa_circ_0076215; hsa_circ_0076216)	≤10^−12^

* In reference [46], PIM1 has been described as an miR-124-3p validated target in astrocytoma.

## Data Availability

The original contributions presented in this study are included in the article/Supplementary Material. Further inquiries can be directed to the corresponding author.

## Supplementary Materials

The following supporting information can be downloaded at: https://www.mdpi.com/article/10.3390/ijms26178263/s1.
