# Supplementary material for: In Silico Identification of circPIM1/miR-16-5p/miR-195-5p/PIM1 Feed-Forward Loop in Recurrent Grade 2 Meningioma
Source: Int J Mol Sci. 2025 Aug 26;26(17):8263. doi: 10.3390/ijms26178263 (PMC12428460; doi:10.3390/ijms26178263)

Figure S1. KEGG pathway enrichment analysis of transcripts positively correlated to PIM1, based on the GSE189672 dataset. Data are extracted from Gene set analysis of the R2 genomics platform and reported in decreasing order of -LOG (p-value): the higher the value the more significant is the enrichment.

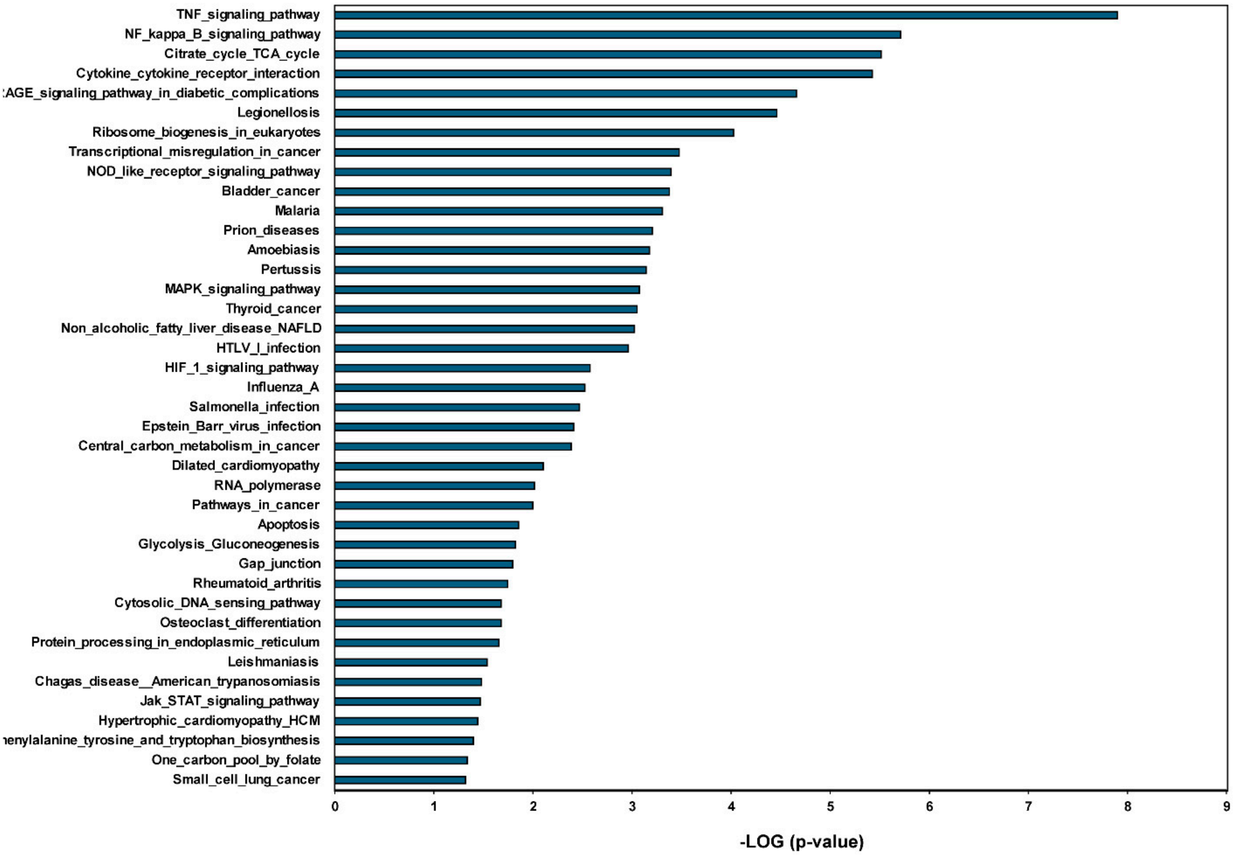

Supplement: Supplementary file 1 [file ijms-26-08263-s001.zip › Figure S1_Rev01_New-190825.pdf]
