# Supplementary material for: In Silico Identification of circPIM1/miR-16-5p/miR-195-5p/PIM1 Feed-Forward Loop in Recurrent Grade 2 Meningioma
Source: Int J Mol Sci. 2025 Aug 26;26(17):8263. doi: 10.3390/ijms26178263 (PMC12428460; doi:10.3390/ijms26178263)

Figure S2. Heatmap showing the expression of candidate MR-miRNAs at the tissue level. As described in the text, MR-miRNA expression was retrieved through the miRNA TissueAtlas 2025.

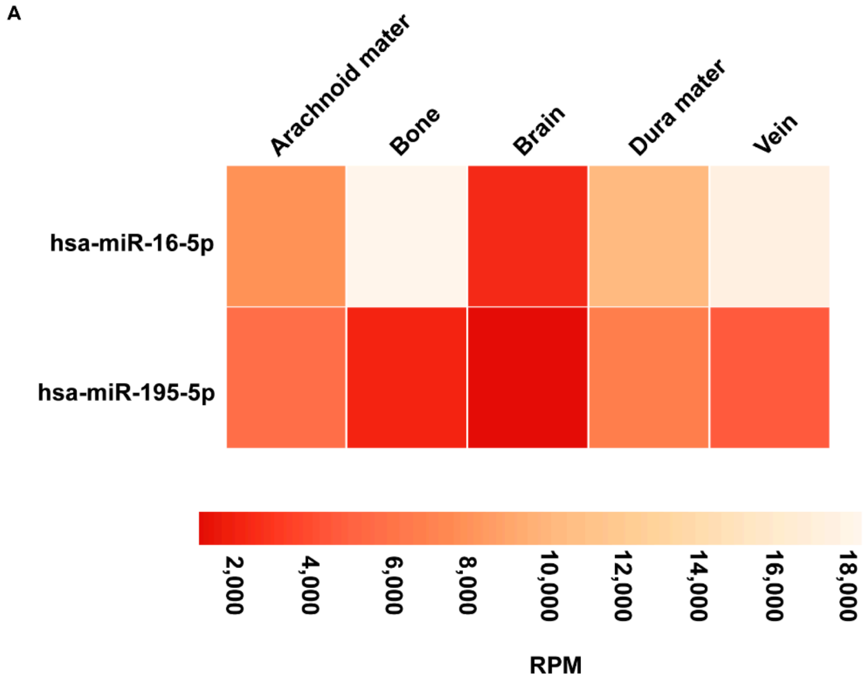

Supplement: Supplementary file 1 [file ijms-26-08263-s001.zip › Figure S2_Rev01.pdf]
