# Supplementary material for: In Silico Identification of circPIM1/miR-16-5p/miR-195-5p/PIM1 Feed-Forward Loop in Recurrent Grade 2 Meningioma
Source: Int J Mol Sci. 2025 Aug 26;26(17):8263. doi: 10.3390/ijms26178263 (PMC12428460; doi:10.3390/ijms26178263)

Figure S4. Volcano plot showing significant higher occurrence of RBP binding sites within MR-circRNAs 0076215 and 0076216 sequences when compared with 139 randomly chosen human circRNAs of similar size.

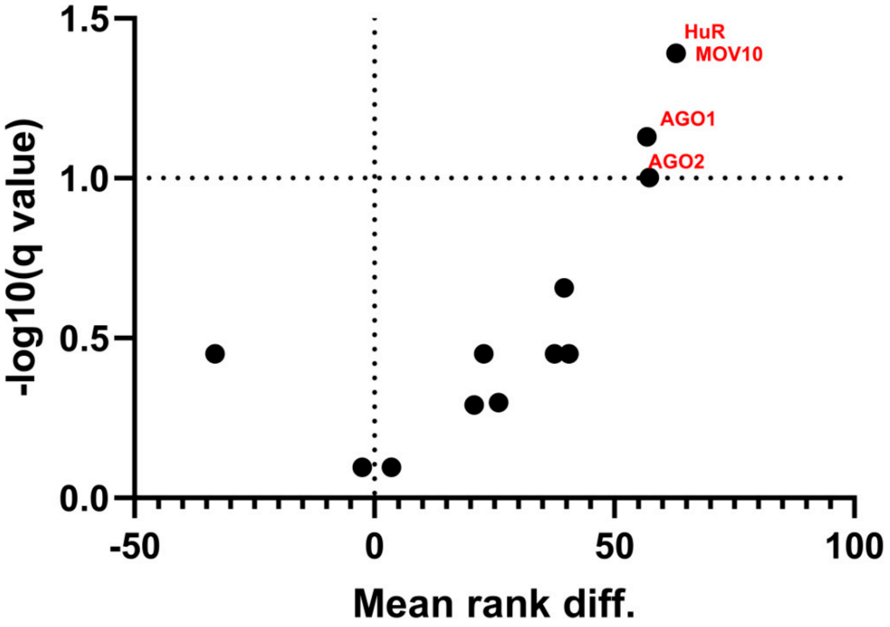

Supplement: Supplementary file 1 [file ijms-26-08263-s001.zip › Figure S4_Rev01.pdf]
