# Supplementary material for: In Silico Identification of circPIM1/miR-16-5p/miR-195-5p/PIM1 Feed-Forward Loop in Recurrent Grade 2 Meningioma
Source: Int J Mol Sci. 2025 Aug 26;26(17):8263. doi: 10.3390/ijms26178263 (PMC12428460; doi:10.3390/ijms26178263)
Supplement: Supplementary file 1 [file ijms-26-08263-s001.zip › Supplementary material-200825_Rev02_corrected.pdf]

Supplementary Material

Our computational analysis, based on the work of Bayley, J. et al. adheres to the inclusion and exclusion criteria outlined in the original dataset [33]. Histopathological diagnosis was conducted according to the 2016 WHO classification criteria, and cases with uncertain diagnoses were excluded. Tumor location was only intracranial and no spinal MNGs were analyzed. Only primary tumors from patients aged 18 years or older were included. WHO grade III MNGs were excluded, as they typically represent recurrent cases. One patient with two anatomically distinct lesions was included with both tumors classified as primary occurrences. All included tumors were from patients without prior surgical or radiation treatment involving the same tumor site, thereby ensuring that the lesions analyzed were truly primary. Only cases with complete and homogeneous clinical data were retained. Cases lacking essential metadata such as age, sex, tumor location, histologic grade, MIB-index, were excluded. A similar principle was applied to biological material quality: tumor samples with inadequate RNA quality were excluded.
