# Supplementary material for: In Silico Identification of circPIM1/miR-16-5p/miR-195-5p/PIM1 Feed-Forward Loop in Recurrent Grade 2 Meningioma
Source: Int J Mol Sci. 2025 Aug 26;26(17):8263. doi: 10.3390/ijms26178263 (PMC12428460; doi:10.3390/ijms26178263)
Supplement: Supplementary file 1 [file ijms-26-08263-s001.zip › Table S1_Rev01.pdf]

Table S1. MiRNAs targeting candidate genes involved in MNG recurrence (MR-miRNAs). MR-miRNAs are ordered based on a decreasing number of targets (i.e.: number of targets for each miRNA among the 34HR-MNG transcripts). MiRNAs targeting at least 4 MR-targets were chosen and reported in this table.

Candidate miRNA	miRNA family	Seed sequence	Number of target genes	Candidate target genes									
hsa-miR-124-3p	MIR-124	AAGGCAC	10	CCND2	CDK6	CDKN2A	CKS2	COL1A1	EZH2	FBLIM1	KIF20A	KRT14	PIM1
hsa-miR-26a-5p	MIR-26	UCAAGUA	7	CCND2	CDK6	CHEK1	CKS2	ESR1	EZH2	PIM1			
hsa-miR-26b-5p	MIR-26	UCAAGUA	7	CCND2	CDK6	CKS2	EZH2	PIM1	TAGLN	TMEM30B			
hsa-miR-16-5p	MIR-15	AGCAGCA	7	CCND2	CDK6	CDKN2A	CHEK1	FGFR4	KDR	PIM1			
hsa-miR-15a-5p	MIR-15	AGCAGCA	6	CCND2	CDK6	CHEK1	FGFR4	MYBL1	PIM1				
hsa-miR-335-5p	MIR-335	CAAGAGC	6	CCND2	FBLIM1	KDR	MMP9	PIM1	TMEM30B				
hsa-miR-15b-5p	MIR-15	AGCAGCA	6	CCND2	CDK6	CHEK1	FGFR4	KDR	PIM1				
hsa-miR-19a-3p	MIR-19	GUGCAAA	5	CCND2	CHEK1	ESR1	FBLIM1	PGK1					
hsa-miR-29b-3p	MIR-29	AGCACCA	5	CCND2	CDK6	COL1A1	ESR1	MMP9					
hsa-let-7a-5p	LET-7	GAGGUAG	5	CCND2	CDK6	EZH2	IGF2	MDM4					
hsa-miR-195-5p	MIR-15	AGCAGCA	5	CCND2	CDK6	CHEK1	FGFR4	PIM1					
hsa-miR-424-5p	MIR-15	AGCAGCA	5	CCND2	CDK6	CHEK1	FGFR4	PIM1					
hsa-miR-497-5p	MIR-15	AGCAGCA	5	CCND2	CDK6	CHEK1	FGFR4	PIM1					

hsa-miR-6838-5p	#N/D	#N/D	5	CCND2	CDK6	CHEK1	FGFR4	PIM1
hsa-miR-19b-3p	MIR-19	GUGCAAA	5	CCND2	CHEK1	ESR1	FBLIM1	PGK1
hsa-miR-193b-3p	MIR-193	ACUGGCC	5	CDK6	CHEK1	ESR1	EZH2	MYBL1
hsa-miR-34a-5p	MIR-34	GGCAGUG	5	CDK6	CDKN2A	GAS1	KDR	MDM4
hsa-miR-320a	MIR-320	AAAGCUG	4	CCND2	CDKN2A	EZH2	IGF2	
hsa-miR-423-5p	MIR-423	GAGGGGC	4	CCND2	CDKN2A	MDM4	TAGLN	
hsa-miR-98-5p	LET-7	GAGGUAG	4	CCND2	EZH2	IFNGR1	MDM4	
hsa-let-7b-5p	LET-7	GAGGUAG	4	CCND2	CDK6	CKS2	MDM4	
hsa-miR-185-5p	MIR-185	GGAGAGA	4	CCND2	CDK6	EZH2	MYBL1	
hsa-miR-548c-3p	#N/D	#N/D	4	CCND2	CDK6	PGK1	SPOP	
hsa-miR-145-5p	MIR-145	UCCAGUU	4	CDK6	ESR1	EZH2	SPOP	
hsa-miR-1236-3p	#N/D	#N/D	4	CDK6	EZH2	KDR	SPOP	
hsa-miR-214-3p	MIR-214	CAGCAGG	4	CDK6	EZH2	FGFR4	PIM1	
hsa-miR-192-5p	MIR-192	UGACCUA	4	CDKN2A	KIF20A	MDM4	PIM1	
hsa-miR-215-5p	MIR-192	UGACCUA	4	CDKN2A	KIF20A	MDM4	PIM1	
hsa-miR-155-5p	MIR-155	UAAUGCU	4	CDKN2A	IFNGR1	KDR	MYBL1	
hsa-miR-24-3p	MIR-24	GGCUCAG	4	CDKN2A	CHEK1	FBLIM1	MDM4	
hsa-miR-9-5p	MIR-9	CUUUGGU	4	ESR1	MDM4	MMP9	TAGLN	

hsa-miR-486-3p	MIR-486	CCUGUAC	4	FGFR4	MDM4	PIM1	TAGLN
