# Supplementary material for: In Silico Identification of circPIM1/miR-16-5p/miR-195-5p/PIM1 Feed-Forward Loop in Recurrent Grade 2 Meningioma
Source: Int J Mol Sci. 2025 Aug 26;26(17):8263. doi: 10.3390/ijms26178263 (PMC12428460; doi:10.3390/ijms26178263)
Supplement: Supplementary file 1 [file ijms-26-08263-s001.zip › Table S2_Rev01.pdf]

Table S2. Pathway enrichment of candidate MR-miRNAs. The top 10 significantly enriched pathways are reported. No targets were found for miR-548c-3p and miR-6838-5p, based on the selected parameters. Data were retrieved through miRPath 4.0.

#	Pathway name	Term Genes	Target Genes (n)	miRNAs (n)	MR-miRNA name	p-value	FDR
1	Pathways in cancer	555	329	29	hsa-let-7a-5p, hsa-let-7b-5p, hsa-miR-124-3p, hsa-miR-145-5p, hsa-miR-155-5p, hsa-miR-15a-5p, hsa-miR-15b-5p, hsa-miR-16-5p, hsa-miR-185-5p, hsa-miR-192-5p, hsa-miR-193b-3p, hsa-miR-195-5p, hsa-miR-19a-3p, hsa-miR-19b-3p, hsa-miR-214-3p, hsa-miR-215-5p, hsa-miR-24-3p, hsa-miR-26a-5p, hsa-miR-26b-5p, hsa-miR-29b-3p, hsa-miR-320a-3p, hsa-miR-335-5p, hsa-miR-34a-5p, hsa-miR-423-5p, hsa-miR-424-5p, hsa-miR-486-3p, hsa-miR-497-5p, hsa-miR-9-5p, hsa-miR-98-5p	$2.44*10^{\wedge-16}$	$7.5*10^{\wedge-14}$
2	Shigellosis	268	179	30	hsa-let-7a-5p, hsa-let-7b-5p, hsa-miR-1236-3p, hsa-miR-124-3p, hsa-miR-145-5p, hsa-miR-155-5p, hsa-miR-15a-5p, hsa-miR-15b-5p, hsa-miR-16-5p, hsa-miR-185-5p, hsa-miR-192-5p, hsa-miR-193b-3p, hsa-miR-195-5p, hsa-miR-19a-3p, hsa-miR-19b-3p, hsa-miR-214-3p, hsa-miR-215-5p, hsa-miR-24-3p, hsa-miR-26a-5p, hsa-miR-26b-5p, hsa-miR-29b-3p, hsa-miR-320a-3p, hsa-miR-335-5p, hsa-miR-34a-5p, hsa-miR-423-5p, hsa-miR-424-5p, hsa-miR-486-3p, hsa-miR-497-5p, hsa-miR-9-5p, hsa-miR-98-5p	$4.4*10^{\wedge-16}$	$7.5*10^{\wedge-14}$
3	Proteoglycans in cancer	220	150	29	hsa-let-7a-5p, hsa-let-7b-5p, hsa-miR-124-3p, hsa-miR-145-5p, hsa-miR-155-5p, hsa-miR-15a-5p, hsa-miR-15b-5p, hsa-miR-16-5p, hsa-miR-185-5p, hsa-miR-192-5p, hsa-miR-193b-3p, hsa-miR-195-5p, hsa-miR-19a-3p, hsa-miR-19b-3p, hsa-miR-214-3p, hsa-miR-215-5p, hsa-miR-24-3p, hsa-miR-26a-5p, hsa-miR-26b-5p, hsa-miR-29b-3p, hsa-miR-320a-3p, hsa-miR-335-5p, hsa-miR-34a-5p, hsa-miR-423-5p, hsa-miR-424-5p, hsa-miR-486-3p, hsa-miR-497-5p, hsa-miR-9-5p, hsa-miR-98-5p	$7.8*10^{\wedge-15}$	$8.86*10^{\wedge-13}$
4	Cell cycle	129	96	29	hsa-let-7a-5p, hsa-let-7b-5p, hsa-miR-124-3p, hsa-miR-145-5p, hsa-miR-155-5p, hsa-miR-15a-5p, hsa-miR-15b-5p, hsa-miR-16-5p, hsa-miR-185-5p, hsa-miR-192-5p, hsa-miR-193b-3p, hsa-miR-195-5p, hsa-miR-19a-3p, hsa-miR-19b-3p, hsa-miR-214-3p, hsa-miR-215-5p, hsa-miR-24-3p, hsa-miR-26a-5p, hsa-miR-26b-5p, hsa-miR-29b-3p, hsa-miR-320a-3p, hsa-	$1.46*10^{\wedge-13}$	$9.96*10^{\wedge-12}$

					miR-335-5p, hsa-miR-34a-5p, hsa-miR-423-5p, hsa-miR-424-5p, hsa-miR-486-3p, hsa-miR-497-5p, hsa-miR-9-5p, hsa-miR-98-5p		
5	Protein processing in endoplasmic reticulum	194	133	29	hsa-let-7a-5p, hsa-let-7b-5p, hsa-miR-124-3p, hsa-miR-145-5p, hsa-miR-155-5p, hsa-miR-15a-5p, hsa-miR-15b-5p, hsa-miR-16-5p, hsa-miR-185-5p, hsa-miR-192-5p, hsa-miR-193b-3p, hsa-miR-195-5p, hsa-miR-19a-3p, hsa-miR-19b-3p, hsa-miR-214-3p, hsa-miR-215-5p, hsa-miR-24-3p, hsa-miR-26a-5p, hsa-miR-26b-5p, hsa-miR-29b-3p, hsa-miR-320a-3p, hsa-miR-335-5p, hsa-miR-34a-5p, hsa-miR-423-5p, hsa-miR-424-5p, hsa-miR-486-3p, hsa-miR-497-5p, hsa-miR-9-5p, hsa-miR-98-5p	$1.4*10^{\wedge -13}$	$9.96*10^{\wedge -12}$
6	Spinocerebellar ataxia	145	105	29	hsa-let-7a-5p, hsa-let-7b-5p, hsa-miR-124-3p, hsa-miR-145-5p, hsa-miR-155-5p, hsa-miR-15a-5p, hsa-miR-15b-5p, hsa-miR-16-5p, hsa-miR-185-5p, hsa-miR-192-5p, hsa-miR-193b-3p, hsa-miR-195-5p, hsa-miR-19a-3p, hsa-miR-19b-3p, hsa-miR-214-3p, hsa-miR-215-5p, hsa-miR-24-3p, hsa-miR-26a-5p, hsa-miR-26b-5p, hsa-miR-29b-3p, hsa-miR-320a-3p, hsa-miR-335-5p, hsa-miR-34a-5p, hsa-miR-423-5p, hsa-miR-424-5p, hsa-miR-486-3p, hsa-miR-497-5p, hsa-miR-9-5p, hsa-miR-98-5p	$2.17*10^{\wedge -13}$	$1.23*10^{\wedge -11}$
7	Ubiquitin mediated proteolysis	142	102	29	hsa-let-7a-5p, hsa-let-7b-5p, hsa-miR-124-3p, hsa-miR-145-5p, hsa-miR-155-5p, hsa-miR-15a-5p, hsa-miR-15b-5p, hsa-miR-16-5p, hsa-miR-185-5p, hsa-miR-192-5p, hsa-miR-193b-3p, hsa-miR-195-5p, hsa-miR-19a-3p, hsa-miR-19b-3p, hsa-miR-214-3p, hsa-miR-215-5p, hsa-miR-24-3p, hsa-miR-26a-5p, hsa-miR-26b-5p, hsa-miR-29b-3p, hsa-miR-320a-3p, hsa-miR-335-5p, hsa-miR-34a-5p, hsa-miR-423-5p, hsa-miR-424-5p, hsa-miR-486-3p, hsa-miR-497-5p, hsa-miR-9-5p, hsa-miR-98-5p	$1.11*10^{\wedge -12}$	$5.39*10^{\wedge -11}$
8	Autophagy - animal	146	104	29	hsa-let-7a-5p, hsa-let-7b-5p, hsa-miR-124-3p, hsa-miR-145-5p, hsa-miR-155-5p, hsa-miR-15a-5p, hsa-miR-15b-5p, hsa-miR-16-5p, hsa-miR-185-5p, hsa-miR-192-5p, hsa-miR-193b-3p, hsa-miR-195-5p, hsa-miR-19a-3p, hsa-miR-19b-3p, hsa-miR-214-3p, hsa-miR-215-5p, hsa-miR-24-3p, hsa-miR-26a-5p, hsa-miR-26b-5p, hsa-miR-29b-3p, hsa-miR-320a-3p, hsa-miR-335-5p, hsa-miR-34a-5p, hsa-miR-423-5p, hsa-miR-424-5p, hsa-miR-486-3p, hsa-miR-497-5p, hsa-miR-9-5p, hsa-miR-98-5p	$1.57*10^{\wedge -12}$	$6.7*10^{\wedge -11}$
9	Renal cell carcinoma	70	58	28	hsa-let-7a-5p, hsa-let-7b-5p, hsa-miR-124-3p, hsa-miR-145-5p, hsa-miR-155-5p, hsa-miR-15a-5p, hsa-miR-15b-5p, hsa-miR-16-5p, hsa-miR-185-5p, hsa-miR-192-5p, hsa-miR-193b-3p, hsa-miR-195-5p, hsa-miR-19a-3p, hsa-miR-19b-3p, hsa-miR-214-3p, hsa-miR-215-5p, hsa-miR-24-3p, hsa-	$4.13*10^{\wedge -12}$	$1.56*10^{\wedge -10}$

					miR-26a-5p, hsa-miR-26b-5p, hsa-miR-29b-3p, hsa-miR-320a-3p, hsa-miR-335-5p, hsa-miR-34a-5p, hsa-miR-423-5p, hsa-miR-424-5p, hsa-miR-497-5p, hsa-miR-9-5p, hsa-miR-98-5p		
10	Pathways of neurodegeneration - multiple diseases	539	306	29	hsa-let-7a-5p, hsa-let-7b-5p, hsa-miR-124-3p, hsa-miR-145-5p, hsa-miR-155-5p, hsa-miR-15a-5p, hsa-miR-15b-5p, hsa-miR-16-5p, hsa-miR-185-5p, hsa-miR-192-5p, hsa-miR-193b-3p, hsa-miR-195-5p, hsa-miR-19a-3p, hsa-miR-19b-3p, hsa-miR-214-3p, hsa-miR-215-5p, hsa-miR-24-3p, hsa-miR-26a-5p, hsa-miR-26b-5p, hsa-miR-29b-3p, hsa-miR-320a-3p, hsa-miR-335-5p, hsa-miR-34a-5p, hsa-miR-423-5p, hsa-miR-424-5p, hsa-miR-486-3p, hsa-miR-497-5p, hsa-miR-9-5p, hsa-miR-98-5p	5.9*10^-12	2.01*10^-10
