# Supplementary material for: In Silico Identification of circPIM1/miR-16-5p/miR-195-5p/PIM1 Feed-Forward Loop in Recurrent Grade 2 Meningioma
Source: Int J Mol Sci. 2025 Aug 26;26(17):8263. doi: 10.3390/ijms26178263 (PMC12428460; doi:10.3390/ijms26178263)
Supplement: Supplementary file 1 [file ijms-26-08263-s001.zip › Table S3_Rev01.pdf]

**Table S3.** Biological process (BPs) enrichment of candidate MR-miRNAs. The top 10 significantly enriched BPs are reported. No targets were found for miR-548c-3p and miR-6838-5p, based on the selected parameters. Data were retrieved through miRPath 4.0.

| # | Biological process (BP) | Term Genes | Target Genes (n) | miRNAs (n) | MR-miRNA name | p-value | FDR |
|---|---|---|---|---|---|---|---|
| 1 | Viral process | 658 | 451 | 29 | hsa-let-7a-5p, hsa-let-7b-5p, hsa-miR-124-3p, hsa-miR-145-5p, hsa-miR-155-5p, hsa-miR-15a-5p, hsa-miR-15b-5p, hsa-miR-16-5p, hsa-miR-185-5p, hsa-miR-192-5p, hsa-miR-193b-3p, hsa-miR-195-5p, hsa-miR-19a-3p, hsa-miR-19b-3p, hsa-miR-214-3p, hsa-miR-215-5p, hsa-miR-24-3p, hsa-miR-26a-5p, hsa-miR-26b-5p, hsa-miR-29b-3p, hsa-miR-320a-3p, hsa-miR-335-5p, hsa-miR-34a-5p, hsa-miR-423-5p, hsa-miR-424-5p, hsa-miR-486-3p, hsa-miR-497-5p, hsa-miR-9-5p, hsa-miR-98-5p | 1.31*10^$^{-47}$ | 1.66*10^$^{-43}$ |
| 2 | Cell cycle | 687 | 453 | 29 | hsa-let-7a-5p, hsa-let-7b-5p, hsa-miR-124-3p, hsa-miR-145-5p, hsa-miR-155-5p, hsa-miR-15a-5p, hsa-miR-15b-5p, hsa-miR-16-5p, hsa-miR-185-5p, hsa-miR-192-5p, hsa-miR-193b-3p, hsa-miR-195-5p, hsa-miR-19a-3p, hsa-miR-19b-3p, hsa-miR-214-3p, hsa-miR-215-5p, hsa-miR-24-3p, hsa-miR-26a-5p, hsa-miR-26b-5p, hsa-miR-29b-3p, hsa-miR-320a-3p, hsa-miR-335-5p, hsa-miR-34a-5p, hsa-miR-423-5p, hsa-miR-424-5p, hsa-miR-486-3p, hsa-miR-497-5p, hsa-miR-9-5p, hsa-miR-98-5p | 5.58*10^$^{-41}$ | 3.52*10^$^{-37}$ |
| 3 | Protein transport | 714 | 459 | 29 | hsa-let-7a-5p, hsa-let-7b-5p, hsa-miR-124-3p, hsa-miR-145-5p, hsa-miR-155-5p, hsa-miR-15a-5p, hsa-miR-15b-5p, hsa-miR-16-5p, hsa-miR-185-5p, hsa-miR-192-5p, hsa-miR-193b-3p, hsa-miR-195-5p, hsa-miR-19a-3p, hsa-miR-19b-3p, hsa-miR-214-3p, hsa-miR-215-5p, hsa-miR-24-3p, hsa-miR-26a-5p, hsa-miR-26b-5p, hsa-miR-29b-3p, hsa-miR-320a-3p, hsa-miR-335-5p, hsa-miR-34a-5p, hsa-miR-423-5p, hsa-miR-424-5p, hsa-miR-486-3p, hsa-miR-497-5p, hsa-miR-9-5p, hsa-miR-98-5p | 2.59*10^$^{-37}$ | 1.09*10^$^{-33}$ |
| 4 | Negative regulation of transcription by RNA polymerase II | 896 | 546 | 29 | hsa-let-7a-5p, hsa-let-7b-5p, hsa-miR-124-3p, hsa-miR-145-5p, hsa-miR-155-5p, hsa-miR-15a-5p, hsa-miR-15b-5p, hsa-miR-16-5p, hsa-miR-185-5p, hsa-miR-192-5p, hsa-miR-193b-3p, hsa-miR-195-5p, hsa-miR-19a-3p, hsa-miR-19b-3p, hsa-miR-214-3p, hsa-miR-215-5p, hsa-miR-24-3p, hsa-miR-26a-5p, hsa-miR-26b-5p, hsa-miR-29b-3p, hsa-miR-320a-3p, hsa- | 9.34*10^$^{-35}$ | 2.94*10^$^{-31}$ |

| | | | | | | | |
|---|---|---|---|---|---|---|---|
| | | | | | miR-335-5p, hsa-miR-34a-5p, hsa-miR-423-5p, hsa-miR-424-5p, hsa-miR-486-3p, hsa-miR-497-5p, hsa-miR-9-5p, hsa-miR-98-5p | | |
| 5 | Chromatin organization | 402 | 279 | 29 | hsa-let-7a-5p, hsa-let-7b-5p, hsa-miR-124-3p, hsa-miR-145-5p, hsa-miR-155-5p, hsa-miR-15a-5p, hsa-miR-15b-5p, hsa-miR-16-5p, hsa-miR-185-5p, hsa-miR-192-5p, hsa-miR-193b-3p, hsa-miR-195-5p, hsa-miR-19a-3p, hsa-miR-19b-3p, hsa-miR-214-3p, hsa-miR-215-5p, hsa-miR-24-3p, hsa-miR-26a-5p, hsa-miR-26b-5p, hsa-miR-29b-3p, hsa-miR-320a-3p, hsa-miR-335-5p, hsa-miR-34a-5p, hsa-miR-423-5p, hsa-miR-424-5p, hsa-miR-486-3p, hsa-miR-497-5p, hsa-miR-9-5p, hsa-miR-98-5p | $3.41*10^{\wedge-31}$ | $8.59*10^{\wedge-28}$ |
| 6 | Positive regulation of transcription, DNA-templated | 663 | 414 | 29 | hsa-let-7a-5p, hsa-let-7b-5p, hsa-miR-124-3p, hsa-miR-145-5p, hsa-miR-155-5p, hsa-miR-15a-5p, hsa-miR-15b-5p, hsa-miR-16-5p, hsa-miR-185-5p, hsa-miR-192-5p, hsa-miR-193b-3p, hsa-miR-195-5p, hsa-miR-19a-3p, hsa-miR-19b-3p, hsa-miR-214-3p, hsa-miR-215-5p, hsa-miR-24-3p, hsa-miR-26a-5p, hsa-miR-26b-5p, hsa-miR-29b-3p, hsa-miR-320a-3p, hsa-miR-335-5p, hsa-miR-34a-5p, hsa-miR-423-5p, hsa-miR-424-5p, hsa-miR-486-3p, hsa-miR-497-5p, hsa-miR-9-5p, hsa-miR-98-5p | $1.13*10^{\wedge-29}$ | $2.38*10^{\wedge-26}$ |
| 7 | Cell division | 428 | 290 | 29 | hsa-let-7a-5p, hsa-let-7b-5p, hsa-miR-124-3p, hsa-miR-145-5p, hsa-miR-155-5p, hsa-miR-15a-5p, hsa-miR-15b-5p, hsa-miR-16-5p, hsa-miR-185-5p, hsa-miR-192-5p, hsa-miR-193b-3p, hsa-miR-195-5p, hsa-miR-19a-3p, hsa-miR-19b-3p, hsa-miR-214-3p, hsa-miR-215-5p, hsa-miR-24-3p, hsa-miR-26a-5p, hsa-miR-26b-5p, hsa-miR-29b-3p, hsa-miR-320a-3p, hsa-miR-335-5p, hsa-miR-34a-5p, hsa-miR-423-5p, hsa-miR-424-5p, hsa-miR-486-3p, hsa-miR-497-5p, hsa-miR-9-5p, hsa-miR-98-5p | $1.48*10^{\wedge-29}$ | $2.66*10^{\wedge-26}$ |
| 8 | Positive regulation of transcription by RNA polymerase II | 1258 | 690 | 29 | hsa-let-7a-5p, hsa-let-7b-5p, hsa-miR-124-3p, hsa-miR-145-5p, hsa-miR-155-5p, hsa-miR-15a-5p, hsa-miR-15b-5p, hsa-miR-16-5p, hsa-miR-185-5p, hsa-miR-192-5p, hsa-miR-193b-3p, hsa-miR-195-5p, hsa-miR-19a-3p, hsa-miR-19b-3p, hsa-miR-214-3p, hsa-miR-215-5p, hsa-miR-24-3p, hsa-miR-26a-5p, hsa-miR-26b-5p, hsa-miR-29b-3p, hsa-miR-320a-3p, hsa-miR-335-5p, hsa-miR-34a-5p, hsa-miR-423-5p, hsa-miR-424-5p, hsa-miR-486-3p, hsa-miR-497-5p, hsa-miR-9-5p, hsa-miR-98-5p | $1.3*10^{\wedge-24}$ | $2.04*10^{\wedge-21}$ |
| 9 | In utero embryonic development | 202 | 151 | 29 | hsa-let-7a-5p, hsa-let-7b-5p, hsa-miR-124-3p, hsa-miR-145-5p, hsa-miR-155-5p, hsa-miR-15a-5p, hsa-miR-15b-5p, hsa-miR-16-5p, hsa-miR-185-5p, hsa-miR-192-5p, hsa-miR-193b-3p, hsa-miR-195-5p, hsa-miR-19a-3p, hsa-miR-19b-3p, hsa-miR-214-3p, hsa-miR-215-5p, hsa-miR-24-3p, hsa- | $1.41*10^{\wedge-22}$ | $1.97*10^{\wedge-19}$ |

| | | | | | | | |
|---|---|---|---|---|---|---|---|
| | | | | | miR-26a-5p, hsa-miR-26b-5p, hsa-miR-29b-3p, hsa-miR-320a-3p, hsa-miR-335-5p, hsa-miR-34a-5p, hsa-miR-423-5p, hsa-miR-424-5p, hsa-miR-486-3p, hsa-miR-497-5p, hsa-miR-9-5p, hsa-miR-98-5p | | |
| 10 | Rhythmic process | 150 | 117 | 29 | hsa-let-7a-5p, hsa-let-7b-5p, hsa-miR-124-3p, hsa-miR-145-5p, hsa-miR-155-5p, hsa-miR-15a-5p, hsa-miR-15b-5p, hsa-miR-16-5p, hsa-miR-185-5p, hsa-miR-192-5p, hsa-miR-193b-3p, hsa-miR-195-5p, hsa-miR-19a-3p, hsa-miR-19b-3p, hsa-miR-214-3p, hsa-miR-215-5p, hsa-miR-24-3p, hsa-miR-26a-5p, hsa-miR-26b-5p, hsa-miR-29b-3p, hsa-miR-320a-3p, hsa-miR-335-5p, hsa-miR-34a-5p, hsa-miR-423-5p, hsa-miR-424-5p, hsa-miR-486-3p, hsa-miR-497-5p, hsa-miR-9-5p, hsa-miR-98-5p | $2.11*10^{\wedge -20}$ | $2.66*10^{\wedge -17}$ |
