# Supplementary material for: In Silico Identification of circPIM1/miR-16-5p/miR-195-5p/PIM1 Feed-Forward Loop in Recurrent Grade 2 Meningioma
Source: Int J Mol Sci. 2025 Aug 26;26(17):8263. doi: 10.3390/ijms26178263 (PMC12428460; doi:10.3390/ijms26178263)
Supplement: Supplementary file 1 [file ijms-26-08263-s001.zip › Table S4_Rev01.pdf]

Table S4. List of circRNAs predicted to sponge MR-miRNAs. Only circRNAs generated by host genes belonging to the 34HR-MNG and targets of MR-miRNAs are shown.

**MR-miRNA**	**CircRNA host's RefSeq RNA**	**CircRNA host's gene name**	**circRNA isoform (circBase ID)**	**Chr position (GRCh38/hg38)**	**clipExpNum**
hsa-miR-124-3p	NM_001759	CCND2	hsa_circ_0025055	chr12:4304725-4304744[+]	2
			hsa_circ_0025057	chr12:4304725-4304744[+]	
			hsa_circ_0025059	chr12:4304725-4304744[+]	
	NM_001145306	CDK6	hsa_circ_0081030	chr7:92613600-92613617[-]	1
	NM_001259	CDK6	hsa_circ_0081031	chr7:92613600-92613617[-]	1
	NM_017556	FBLIM1	hsa_circ_0010089	chr1:15785028-15785047[+]	1
			hsa_circ_0010090	chr1:15785028-15785047[+]	
			hsa_circ_0010091	chr1:15785028-15785047[+]	
			hsa_circ_0010092	chr1:15785028-15785047[+]	
			hsa_circ_0010099	chr1:15785028-15785047[+]	
			hsa_circ_0010101	chr1:15785028-15785047[+]	
			hsa_circ_0010102	chr1:15785028-15785047[+]	
			hsa_circ_0010103	chr1:15785028-15785047[+]	
	NM_001243186	PIM1	hsa_circ_0076213	chr6:37174520-37174539[+]	4
	NM_002648	PIM1	hsa_circ_0076214	chr6:37174520-37174539[+]	4
			hsa_circ_0076215	chr6:37174520-37174539[+]	
			hsa_circ_0076216	chr6:37174520-37174539[+]	
hsa-miR-26a-5p	NM_001759	CCND2	hsa_circ_0025055	chr12:4301393-4301413[+]	11

			hsa_circ_0025057	chr12:4301393-4301413[+]	
			hsa_circ_0025059	chr12:4301393-4301413[+]	
	NM_001145306	CDK6	hsa_circ_0081030	chr7:92608045-92608066[-]	5
	NM_001259	CDK6	hsa_circ_0081031	chr7:92608045-92608066[-]	5
	NM_001145306	CDK6	hsa_circ_0081030	chr7:92606115-92606136[-]	3
	NM_001259	CDK6	hsa_circ_0081031	chr7:92606115-92606136[-]	3
	NM_001827	CKS2	hsa_circ_0087441	chr9:89316482-89316504[+]	2
			hsa_circ_0087442	chr9:89316482-89316504[+]	
	NM_001243186	PIM1	hsa_circ_0076213	chr6:37174551-37174576[+]	12
	NM_002648	PIM1	hsa_circ_0076214	chr6:37174551-37174576[+]	12
			hsa_circ_0076215	chr6:37174551-37174576[+]	
			hsa_circ_0076216	chr6:37174551-37174576[+]	
hsa-miR-26b-5p	NM_001759	CCND2	hsa_circ_0025055	chr12:4301394-4301413[+]	11
			hsa_circ_0025057	chr12:4301394-4301413[+]	
			hsa_circ_0025059	chr12:4301394-4301413[+]	
	NM_001145306	CDK6	hsa_circ_0081030	chr7:92608045-92608065[-]	5
	NM_001259	CDK6	hsa_circ_0081031	chr7:92608045-92608065[-]	5
	NM_001145306	CDK6	hsa_circ_0081030	chr7:92606115-92606135[-]	3
	NM_001259	CDK6	hsa_circ_0081031	chr7:92606115-92606135[-]	3
	NM_001827	CKS2	hsa_circ_0087441	chr9:89316483-89316504[+]	2
			hsa_circ_0087442	chr9:89316483-89316504[+]	

	NM_001243186	PIM1	hsa_circ_0076213	chr6:37174552-37174576[+]	12
	NM_002648	PIM1	hsa_circ_0076214	chr6:37174552-37174576[+]	12
			hsa_circ_0076215	chr6:37174552-37174576[+]	
			hsa_circ_0076216	chr6:37174552-37174576[+]	
hsa-miR-16-5p	NM_001759	CCND2	hsa_circ_0025055	chr12:4301753-4301780[+]	10
			hsa_circ_0025057	chr12:4301753-4301780[+]	
			hsa_circ_0025059	chr12:4301753-4301780[+]	
	NM_001145306	CDK6	hsa_circ_0081030	chr7:92608750-92608770[-]	6
	NM_001259	CDK6	hsa_circ_0081031	chr7:92608750-92608770[-]	6
	NM_001145306	CDK6	hsa_circ_0081030	chr7:92609660-92609683[-]	2
	NM_001259	CDK6	hsa_circ_0081031	chr7:92609660-92609683[-]	2
	NM_001114121	CHEK1	hsa_circ_0024791	chr11:125655343-125655365[+]	4
			hsa_circ_0024794	chr11:125655343-125655365[+]	
	NM_001114122	CHEK1	hsa_circ_0024793	chr11:125655343-125655365[+]	4
	NM_213647	FGFR4	hsa_circ_0075147	chr5:177093217-177093238[+]	2
	NM_001243186	PIM1	hsa_circ_0076213	chr6:37174578-37174599[+]	12
	NM_002648	PIM1	hsa_circ_0076214	chr6:37174578-37174599[+]	12
			hsa_circ_0076215	chr6:37174578-37174599[+]	
			hsa_circ_0076216	chr6:37174578-37174599[+]	
hsa-miR-15a-5p	NM_001759	CCND2	hsa_circ_0025055	chr12:4301759-4301780[+]	10
			hsa_circ_0025057	chr12:4301759-4301780[+]	

			hsa_circ_0025059	chr12:4301759-4301780[+]	
	NM_001145306	CDK6	hsa_circ_0081030	chr7:92608750-92608774[-]	6
	NM_001259	CDK6	hsa_circ_0081031	chr7:92608750-92608774[-]	6
	NM_001145306	CDK6	hsa_circ_0081030	chr7:92609660-92609680[-]	2
	NM_001259	CDK6	hsa_circ_0081031	chr7:92609660-92609680[-]	2
	NM_001114121	CHEK1	hsa_circ_0024791	chr11:125655343-125655365[+]	4
			hsa_circ_0024794	chr11:125655343-125655365[+]	
	NM_001114122	CHEK1	hsa_circ_0024793	chr11:125655343-125655365[+]	4
	NM_213647	FGFR4	hsa_circ_0075147	chr5:177093216-177093238[+]	2
	NM_001080416	MYBL1	hsa_circ_0084640	chr8:66563226-66563247[-]	4
	NM_001080416	MYBL1	hsa_circ_0084640	chr8:66563255-66563276[-]	1
	NM_001243186	PIM1	hsa_circ_0076213	chr6:37174578-37174599[+]	12
	NM_002648	PIM1	hsa_circ_0076214	chr6:37174578-37174599[+]	12
			hsa_circ_0076215	chr6:37174578-37174599[+]	
			hsa_circ_0076216	chr6:37174578-37174599[+]	
hsa-miR-335-5p	NM_017556	FBLIM1	hsa_circ_0010089	chr1:15784722-15784745[+]	3
			hsa_circ_0010090	chr1:15784722-15784745[+]	
			hsa_circ_0010091	chr1:15784722-15784745[+]	
			hsa_circ_0010092	chr1:15784722-15784745[+]	
			hsa_circ_0010099	chr1:15784722-15784745[+]	
			hsa_circ_0010101	chr1:15784722-15784745[+]	

			hsa_circ_0010102	chr1:15784722-15784745[+]	
			hsa_circ_0010103	chr1:15784722-15784745[+]	
hsa-miR-15b-5p	NM_001759	CCND2	hsa_circ_0025055	chr12:4301759-4301780[+]	10
			hsa_circ_0025057	chr12:4301759-4301780[+]	
			hsa_circ_0025059	chr12:4301759-4301780[+]	
	NM_001145306	CDK6	hsa_circ_0081030	chr7:92608750-92608774[-]	6
	NM_001259	CDK6	hsa_circ_0081031	chr7:92608750-92608774[-]	6
	NM_001145306	CDK6	hsa_circ_0081030	chr7:92609660-92609680[-]	2
	NM_001259	CDK6	hsa_circ_0081031	chr7:92609660-92609680[-]	2
	NM_001114121	CHEK1	hsa_circ_0024791	chr11:125655347-125655365[+]	3
			hsa_circ_0024794	chr11:125655347-125655365[+]	
	NM_001114122	CHEK1	hsa_circ_0024793	chr11:125655347-125655365[+]	3
	NM_213647	FGFR4	hsa_circ_0075147	chr5:177093216-177093238[+]	2
	NM_001243186	PIM1	hsa_circ_0076213	chr6:37174578-37174599[+]	12
	NM_002648	PIM1	hsa_circ_0076214	chr6:37174578-37174599[+]	12
			hsa_circ_0076215	chr6:37174578-37174599[+]	
			hsa_circ_0076216	chr6:37174578-37174599[+]	
hsa-miR-19a-3p	NM_001759	CCND2	hsa_circ_0025055	chr12:4302312-4302332[+]	15
			hsa_circ_0025057	chr12:4302312-4302332[+]	
			hsa_circ_0025059	chr12:4302312-4302332[+]	
	NM_001759	CCND2	hsa_circ_0025055	chr12:4301471-4301493[+]	10

			hsa_circ_0025057	chr12:4301471-4301493[+]	
			hsa_circ_0025059	chr12:4301471-4301493[+]	
	NM_001114122	CHEK1	hsa_circ_0024793	chr11:125657004-125657028[+]	21
hsa-miR-29b-3p	NM_001759	CCND2	hsa_circ_0025055	chr12:4302553-4302575[+]	13
			hsa_circ_0025057	chr12:4302553-4302575[+]	
			hsa_circ_0025059	chr12:4302553-4302575[+]	
	NM_001759	CCND2	hsa_circ_0025055	chr12:4300903-4300922[+]	5
			hsa_circ_0025057	chr12:4300903-4300922[+]	
			hsa_circ_0025059	chr12:4300903-4300922[+]	
	NM_001759	CCND2	hsa_circ_0025054	chr12:4278760-4278780[+]	1
			hsa_circ_0025055	chr12:4278760-4278780[+]	
			hsa_circ_0025056	chr12:4278760-4278780[+]	
			hsa_circ_0025057	chr12:4278760-4278780[+]	
			hsa_circ_0025058	chr12:4278760-4278780[+]	
			hsa_circ_0025059	chr12:4278760-4278780[+]	
	NM_001145306	CDK6	hsa_circ_0081030	chr7:92606426-92606451[-]	12
	NM_001259	CDK6	hsa_circ_0081031	chr7:92606426-92606451[-]	12
	NM_001145306	CDK6	hsa_circ_0081030	chr7:92605995-92606017[-]	3
	NM_001259	CDK6	hsa_circ_0081031	chr7:92605995-92606017[-]	3
	NM_000088	COL1A1	hsa_circ_0044504	chr17:50190057-50190078[-]	17
			hsa_circ_0044505	chr17:50190057-50190078[-]	

	hsa_circ_0044506	chr17:50190057-50190078[-]	
	hsa_circ_0044507	chr17:50190057-50190078[-]	
	hsa_circ_0044508	chr17:50190057-50190078[-]	
	hsa_circ_0044509	chr17:50190057-50190078[-]	
	hsa_circ_0044510	chr17:50190057-50190078[-]	
	hsa_circ_0044511	chr17:50190057-50190078[-]	
	hsa_circ_0044512	chr17:50190057-50190078[-]	
	hsa_circ_0044514	chr17:50190057-50190078[-]	
	hsa_circ_0044515	chr17:50190057-50190078[-]	
	hsa_circ_0044516	chr17:50190057-50190078[-]	
	hsa_circ_0044517	chr17:50190057-50190078[-]	
	hsa_circ_0044518	chr17:50190057-50190078[-]	
	hsa_circ_0044519	chr17:50190057-50190078[-]	
	hsa_circ_0044520	chr17:50190057-50190078[-]	
	hsa_circ_0044521	chr17:50190057-50190078[-]	
	hsa_circ_0044522	chr17:50190057-50190078[-]	
	hsa_circ_0044523	chr17:50190057-50190078[-]	
	hsa_circ_0044524	chr17:50190057-50190078[-]	
	hsa_circ_0044525	chr17:50190057-50190078[-]	
	hsa_circ_0044526	chr17:50190057-50190078[-]	
	hsa_circ_0044527	chr17:50190057-50190078[-]	

	hsa_circ_0044528	chr17:50190057-50190078[-]
	hsa_circ_0044529	chr17:50190057-50190078[-]
	hsa_circ_0044530	chr17:50190057-50190078[-]
	hsa_circ_0044531	chr17:50190057-50190078[-]
	hsa_circ_0044532	chr17:50190057-50190078[-]
	hsa_circ_0044533	chr17:50190057-50190078[-]
	hsa_circ_0044534	chr17:50190057-50190078[-]
	hsa_circ_0044535	chr17:50190057-50190078[-]
	hsa_circ_0044536	chr17:50190057-50190078[-]
	hsa_circ_0044537	chr17:50190057-50190078[-]
	hsa_circ_0044538	chr17:50190057-50190078[-]
	hsa_circ_0044539	chr17:50190057-50190078[-]
	hsa_circ_0044540	chr17:50190057-50190078[-]
	hsa_circ_0044541	chr17:50190057-50190078[-]
	hsa_circ_0044542	chr17:50190057-50190078[-]
	hsa_circ_0044543	chr17:50190057-50190078[-]
	hsa_circ_0044544	chr17:50190057-50190078[-]
	hsa_circ_0044545	chr17:50190057-50190078[-]
	hsa_circ_0044546	chr17:50190057-50190078[-]
	hsa_circ_0044547	chr17:50190057-50190078[-]
	hsa_circ_0044548	chr17:50190057-50190078[-]

			hsa_circ_0044549	chr17:50190057-50190078[-]	
	NM_000088	COL1A1	hsa_circ_0044504	chr17:50190084-50190108[-]	11
			hsa_circ_0044505	chr17:50190084-50190108[-]	
			hsa_circ_0044506	chr17:50190084-50190108[-]	
			hsa_circ_0044507	chr17:50190084-50190108[-]	
			hsa_circ_0044508	chr17:50190084-50190108[-]	
			hsa_circ_0044509	chr17:50190084-50190108[-]	
			hsa_circ_0044510	chr17:50190084-50190108[-]	
			hsa_circ_0044511	chr17:50190084-50190108[-]	
			hsa_circ_0044512	chr17:50190084-50190108[-]	
			hsa_circ_0044514	chr17:50190084-50190108[-]	
			hsa_circ_0044515	chr17:50190084-50190108[-]	
			hsa_circ_0044516	chr17:50190084-50190108[-]	
			hsa_circ_0044517	chr17:50190084-50190108[-]	
			hsa_circ_0044518	chr17:50190084-50190108[-]	
			hsa_circ_0044519	chr17:50190084-50190108[-]	
			hsa_circ_0044520	chr17:50190084-50190108[-]	
			hsa_circ_0044521	chr17:50190084-50190108[-]	
			hsa_circ_0044522	chr17:50190084-50190108[-]	
			hsa_circ_0044523	chr17:50190084-50190108[-]	
			hsa_circ_0044524	chr17:50190084-50190108[-]	

	hsa_circ_0044525	chr17:50190084-50190108[-]
	hsa_circ_0044526	chr17:50190084-50190108[-]
	hsa_circ_0044527	chr17:50190084-50190108[-]
	hsa_circ_0044528	chr17:50190084-50190108[-]
	hsa_circ_0044529	chr17:50190084-50190108[-]
	hsa_circ_0044530	chr17:50190084-50190108[-]
	hsa_circ_0044531	chr17:50190084-50190108[-]
	hsa_circ_0044532	chr17:50190084-50190108[-]
	hsa_circ_0044533	chr17:50190084-50190108[-]
	hsa_circ_0044534	chr17:50190084-50190108[-]
	hsa_circ_0044535	chr17:50190084-50190108[-]
	hsa_circ_0044536	chr17:50190084-50190108[-]
	hsa_circ_0044537	chr17:50190084-50190108[-]
	hsa_circ_0044538	chr17:50190084-50190108[-]
	hsa_circ_0044539	chr17:50190084-50190108[-]
	hsa_circ_0044540	chr17:50190084-50190108[-]
	hsa_circ_0044541	chr17:50190084-50190108[-]
	hsa_circ_0044542	chr17:50190084-50190108[-]
	hsa_circ_0044543	chr17:50190084-50190108[-]
	hsa_circ_0044544	chr17:50190084-50190108[-]
	hsa_circ_0044545	chr17:50190084-50190108[-]

			hsa_circ_0044546	chr17:50190084-50190108[-]	
			hsa_circ_0044547	chr17:50190084-50190108[-]	
			hsa_circ_0044548	chr17:50190084-50190108[-]	
			hsa_circ_0044549	chr17:50190084-50190108[-]	
	NM_000088	COL1A1	hsa_circ_0044510	chr17:50196166-50196190[-]	7
			hsa_circ_0044511	chr17:50196166-50196190[-]	
			hsa_circ_0044512	chr17:50196166-50196190[-]	
			hsa_circ_0044518	chr17:50196166-50196190[-]	
			hsa_circ_0044519	chr17:50196166-50196190[-]	
			hsa_circ_0044522	chr17:50196166-50196190[-]	
			hsa_circ_0044524	chr17:50196166-50196190[-]	
			hsa_circ_0044527	chr17:50196166-50196190[-]	
			hsa_circ_0044528	chr17:50196166-50196190[-]	
			hsa_circ_0044529	chr17:50196166-50196190[-]	
			hsa_circ_0044535	chr17:50196166-50196190[-]	
			hsa_circ_0044536	chr17:50196166-50196190[-]	
			hsa_circ_0044539	chr17:50196166-50196190[-]	
			hsa_circ_0044543	chr17:50196166-50196190[-]	
			hsa_circ_0044548	chr17:50196166-50196190[-]	
			hsa_circ_0044549	chr17:50196166-50196190[-]	
			hsa_circ_0044551	chr17:50196166-50196190[-]	

			hsa_circ_0044552	chr17:50196166-50196190[-]	
			hsa_circ_0044553	chr17:50196166-50196190[-]	
			hsa_circ_0044554	chr17:50196166-50196190[-]	
			hsa_circ_0044555	chr17:50196166-50196190[-]	
			hsa_circ_0044557	chr17:50196166-50196190[-]	
			hsa_circ_0044558	chr17:50196166-50196190[-]	
			hsa_circ_0044559	chr17:50196166-50196190[-]	
			hsa_circ_0044560	chr17:50196166-50196190[-]	
			hsa_circ_0044561	chr17:50196166-50196190[-]	
			hsa_circ_0044562	chr17:50196166-50196190[-]	
			hsa_circ_0044563	chr17:50196166-50196190[-]	
	NM_000088	COL1A1	hsa_circ_0044509	chr17:50195578-50195599[-]	7
			hsa_circ_0044510	chr17:50195578-50195599[-]	
			hsa_circ_0044511	chr17:50195578-50195599[-]	
			hsa_circ_0044512	chr17:50195578-50195599[-]	
			hsa_circ_0044518	chr17:50195578-50195599[-]	
			hsa_circ_0044519	chr17:50195578-50195599[-]	
			hsa_circ_0044520	chr17:50195578-50195599[-]	
			hsa_circ_0044522	chr17:50195578-50195599[-]	
			hsa_circ_0044524	chr17:50195578-50195599[-]	
			hsa_circ_0044526	chr17:50195578-50195599[-]	

	hsa_circ_0044527	chr17:50195578-50195599[-]
	hsa_circ_0044528	chr17:50195578-50195599[-]
	hsa_circ_0044529	chr17:50195578-50195599[-]
	hsa_circ_0044534	chr17:50195578-50195599[-]
	hsa_circ_0044535	chr17:50195578-50195599[-]
	hsa_circ_0044536	chr17:50195578-50195599[-]
	hsa_circ_0044539	chr17:50195578-50195599[-]
	hsa_circ_0044543	chr17:50195578-50195599[-]
	hsa_circ_0044547	chr17:50195578-50195599[-]
	hsa_circ_0044548	chr17:50195578-50195599[-]
	hsa_circ_0044549	chr17:50195578-50195599[-]
	hsa_circ_0044551	chr17:50195578-50195599[-]
	hsa_circ_0044552	chr17:50195578-50195599[-]
	hsa_circ_0044553	chr17:50195578-50195599[-]
	hsa_circ_0044554	chr17:50195578-50195599[-]
	hsa_circ_0044555	chr17:50195578-50195599[-]
	hsa_circ_0044557	chr17:50195578-50195599[-]
	hsa_circ_0044558	chr17:50195578-50195599[-]
	hsa_circ_0044559	chr17:50195578-50195599[-]
	hsa_circ_0044560	chr17:50195578-50195599[-]
	hsa_circ_0044561	chr17:50195578-50195599[-]

	NM_000088	COL1A1	hsa_circ_0044502	chr17:50184572-50184593[-]	6
			hsa_circ_0044503	chr17:50184572-50184593[-]	
			hsa_circ_0044504	chr17:50184572-50184593[-]	
			hsa_circ_0044505	chr17:50184572-50184593[-]	
			hsa_circ_0044506	chr17:50184572-50184593[-]	
			hsa_circ_0044507	chr17:50184572-50184593[-]	
			hsa_circ_0044508	chr17:50184572-50184593[-]	
			hsa_circ_0044509	chr17:50184572-50184593[-]	
			hsa_circ_0044510	chr17:50184572-50184593[-]	
			hsa_circ_0044511	chr17:50184572-50184593[-]	
			hsa_circ_0044512	chr17:50184572-50184593[-]	
	NM_000088	COL1A1	hsa_circ_0044502	chr17:50184572-50184593[-]	6
			hsa_circ_0044503	chr17:50184572-50184593[-]	
			hsa_circ_0044504	chr17:50184572-50184593[-]	
			hsa_circ_0044505	chr17:50184572-50184593[-]	
			hsa_circ_0044506	chr17:50184572-50184593[-]	
			hsa_circ_0044507	chr17:50184572-50184593[-]	
			hsa_circ_0044508	chr17:50184572-50184593[-]	
			hsa_circ_0044509	chr17:50184572-50184593[-]	
			hsa_circ_0044510	chr17:50184572-50184593[-]	
			hsa_circ_0044511	chr17:50184572-50184593[-]	

			hsa_circ_0044512	chr17:50184572-50184593[-]	
	NM_000088	COL1A1	hsa_circ_0044509	chr17:50195463-50195478[-]	5
			hsa_circ_0044510	chr17:50195463-50195478[-]	
			hsa_circ_0044511	chr17:50195463-50195478[-]	
			hsa_circ_0044512	chr17:50195463-50195478[-]	
			hsa_circ_0044518	chr17:50195463-50195478[-]	
			hsa_circ_0044519	chr17:50195463-50195478[-]	
			hsa_circ_0044520	chr17:50195463-50195478[-]	
			hsa_circ_0044522	chr17:50195463-50195478[-]	
			hsa_circ_0044524	chr17:50195463-50195478[-]	
			hsa_circ_0044526	chr17:50195463-50195478[-]	
			hsa_circ_0044527	chr17:50195463-50195478[-]	
			hsa_circ_0044528	chr17:50195463-50195478[-]	
			hsa_circ_0044529	chr17:50195463-50195478[-]	
			hsa_circ_0044533	chr17:50195463-50195478[-]	
			hsa_circ_0044534	chr17:50195463-50195478[-]	
			hsa_circ_0044535	chr17:50195463-50195478[-]	
			hsa_circ_0044536	chr17:50195463-50195478[-]	
			hsa_circ_0044539	chr17:50195463-50195478[-]	
			hsa_circ_0044543	chr17:50195463-50195478[-]	
			hsa_circ_0044547	chr17:50195463-50195478[-]	

			hsa_circ_0044548	chr17:50195463-50195478[-]	
			hsa_circ_0044549	chr17:50195463-50195478[-]	
			hsa_circ_0044551	chr17:50195463-50195478[-]	
			hsa_circ_0044552	chr17:50195463-50195478[-]	
			hsa_circ_0044553	chr17:50195463-50195478[-]	
			hsa_circ_0044554	chr17:50195463-50195478[-]	
			hsa_circ_0044555	chr17:50195463-50195478[-]	
			hsa_circ_0044557	chr17:50195463-50195478[-]	
			hsa_circ_0044558	chr17:50195463-50195478[-]	
			hsa_circ_0044559	chr17:50195463-50195478[-]	
			hsa_circ_0044560	chr17:50195463-50195478[-]	
			hsa_circ_0044561	chr17:50195463-50195478[-]	
	NM_000088	COL1A1	hsa_circ_0044504	chr17:50189388-50189410[-]	4
			hsa_circ_0044505	chr17:50189388-50189410[-]	
			hsa_circ_0044506	chr17:50189388-50189410[-]	
			hsa_circ_0044507	chr17:50189388-50189410[-]	
			hsa_circ_0044508	chr17:50189388-50189410[-]	
			hsa_circ_0044509	chr17:50189388-50189410[-]	
			hsa_circ_0044510	chr17:50189388-50189410[-]	
			hsa_circ_0044511	chr17:50189388-50189410[-]	
			hsa_circ_0044512	chr17:50189388-50189410[-]	

		hsa_circ_0044514	chr17:50189388-50189410[-]
		hsa_circ_0044515	chr17:50189388-50189410[-]
		hsa_circ_0044516	chr17:50189388-50189410[-]
		hsa_circ_0044517	chr17:50189388-50189410[-]
		hsa_circ_0044518	chr17:50189388-50189410[-]
		hsa_circ_0044519	chr17:50189388-50189410[-]
		hsa_circ_0044520	chr17:50189388-50189410[-]
		hsa_circ_0044521	chr17:50189388-50189410[-]
		hsa_circ_0044522	chr17:50189388-50189410[-]
		hsa_circ_0044523	chr17:50189388-50189410[-]
		hsa_circ_0044524	chr17:50189388-50189410[-]
		hsa_circ_0044525	chr17:50189388-50189410[-]
		hsa_circ_0044526	chr17:50189388-50189410[-]
		hsa_circ_0044527	chr17:50189388-50189410[-]
		hsa_circ_0044528	chr17:50189388-50189410[-]
		hsa_circ_0044529	chr17:50189388-50189410[-]
		hsa_circ_0044530	chr17:50189388-50189410[-]
		hsa_circ_0044531	chr17:50189388-50189410[-]
		hsa_circ_0044532	chr17:50189388-50189410[-]
		hsa_circ_0044533	chr17:50189388-50189410[-]
		hsa_circ_0044534	chr17:50189388-50189410[-]

			hsa_circ_0044535	chr17:50189388-50189410[-]	
			hsa_circ_0044536	chr17:50189388-50189410[-]	
			hsa_circ_0044537	chr17:50189388-50189410[-]	
			hsa_circ_0044538	chr17:50189388-50189410[-]	
			hsa_circ_0044539	chr17:50189388-50189410[-]	
	NM_000088	COL1A1	hsa_circ_0044504	chr17:50188586-50188607[-]	3
			hsa_circ_0044505	chr17:50188586-50188607[-]	
			hsa_circ_0044506	chr17:50188586-50188607[-]	
			hsa_circ_0044507	chr17:50188586-50188607[-]	
			hsa_circ_0044508	chr17:50188586-50188607[-]	
			hsa_circ_0044509	chr17:50188586-50188607[-]	
			hsa_circ_0044510	chr17:50188586-50188607[-]	
			hsa_circ_0044511	chr17:50188586-50188607[-]	
			hsa_circ_0044512	chr17:50188586-50188607[-]	
			hsa_circ_0044514	chr17:50188586-50188607[-]	
			hsa_circ_0044515	chr17:50188586-50188607[-]	
			hsa_circ_0044516	chr17:50188586-50188607[-]	
			hsa_circ_0044517	chr17:50188586-50188607[-]	
			hsa_circ_0044518	chr17:50188586-50188607[-]	
			hsa_circ_0044519	chr17:50188586-50188607[-]	
			hsa_circ_0044520	chr17:50188586-50188607[-]	

			hsa_circ_0044521	chr17:50188586-50188607[-]	
			hsa_circ_0044522	chr17:50188586-50188607[-]	
			hsa_circ_0044523	chr17:50188586-50188607[-]	
			hsa_circ_0044524	chr17:50188586-50188607[-]	
			hsa_circ_0044525	chr17:50188586-50188607[-]	
			hsa_circ_0044526	chr17:50188586-50188607[-]	
	NM_000088	COL1A1	hsa_circ_0044509	chr17:50195306-50195327[-]	2
			hsa_circ_0044510	chr17:50195306-50195327[-]	
			hsa_circ_0044511	chr17:50195306-50195327[-]	
			hsa_circ_0044512	chr17:50195306-50195327[-]	
			hsa_circ_0044517	chr17:50195306-50195327[-]	
			hsa_circ_0044518	chr17:50195306-50195327[-]	
			hsa_circ_0044519	chr17:50195306-50195327[-]	
			hsa_circ_0044520	chr17:50195306-50195327[-]	
			hsa_circ_0044522	chr17:50195306-50195327[-]	
			hsa_circ_0044524	chr17:50195306-50195327[-]	
			hsa_circ_0044526	chr17:50195306-50195327[-]	
			hsa_circ_0044527	chr17:50195306-50195327[-]	
			hsa_circ_0044528	chr17:50195306-50195327[-]	
			hsa_circ_0044529	chr17:50195306-50195327[-]	
			hsa_circ_0044533	chr17:50195306-50195327[-]	

			hsa_circ_0044534	chr17:50195306-50195327[-]	
			hsa_circ_0044535	chr17:50195306-50195327[-]	
			hsa_circ_0044536	chr17:50195306-50195327[-]	
			hsa_circ_0044539	chr17:50195306-50195327[-]	
			hsa_circ_0044543	chr17:50195306-50195327[-]	
			hsa_circ_0044547	chr17:50195306-50195327[-]	
			hsa_circ_0044548	chr17:50195306-50195327[-]	
			hsa_circ_0044549	chr17:50195306-50195327[-]	
			hsa_circ_0044551	chr17:50195306-50195327[-]	
			hsa_circ_0044552	chr17:50195306-50195327[-]	
			hsa_circ_0044553	chr17:50195306-50195327[-]	
			hsa_circ_0044554	chr17:50195306-50195327[-]	
			hsa_circ_0044555	chr17:50195306-50195327[-]	
			hsa_circ_0044557	chr17:50195306-50195327[-]	
			hsa_circ_0044558	chr17:50195306-50195327[-]	
			hsa_circ_0044559	chr17:50195306-50195327[-]	
			hsa_circ_0044560	chr17:50195306-50195327[-]	
			hsa_circ_0044561	chr17:50195306-50195327[-]	
	NM_000088	COL1A1	hsa_circ_0044503	chr17:50187098-50187119[-]	1
			hsa_circ_0044504	chr17:50187098-50187119[-]	
			hsa_circ_0044505	chr17:50187098-50187119[-]	

			hsa_circ_0044506	chr17:50187098-50187119[-]	
			hsa_circ_0044507	chr17:50187098-50187119[-]	
			hsa_circ_0044508	chr17:50187098-50187119[-]	
			hsa_circ_0044509	chr17:50187098-50187119[-]	
			hsa_circ_0044510	chr17:50187098-50187119[-]	
			hsa_circ_0044511	chr17:50187098-50187119[-]	
			hsa_circ_0044512	chr17:50187098-50187119[-]	
			hsa_circ_0044513	chr17:50187098-50187119[-]	
			hsa_circ_0044514	chr17:50187098-50187119[-]	
			hsa_circ_0044515	chr17:50187098-50187119[-]	
			hsa_circ_0044516	chr17:50187098-50187119[-]	
			hsa_circ_0044517	chr17:50187098-50187119[-]	
			hsa_circ_0044518	chr17:50187098-50187119[-]	
			hsa_circ_0044519	chr17:50187098-50187119[-]	
			hsa_circ_0044520	chr17:50187098-50187119[-]	
hsa-let-7a-5p	NM_002393	MDM4	hsa_circ_0016156	chr1:204557032-204557053[+]	18
			hsa_circ_0016160	chr1:204557032-204557053[+]	
			hsa_circ_0016162	chr1:204557032-204557053[+]	
			hsa_circ_0016164	chr1:204557032-204557053[+]	
			hsa_circ_0016165	chr1:204557032-204557053[+]	
			hsa_circ_0016166	chr1:204557032-204557053[+]	

			hsa_circ_0016167	chr1:204557032-204557053[+]	
	NM_002393	MDM4	hsa_circ_0016156	chr1:204538256-204538280[+]	7
			hsa_circ_0016159	chr1:204538256-204538280[+]	
			hsa_circ_0016160	chr1:204538256-204538280[+]	
			hsa_circ_0016162	chr1:204538256-204538280[+]	
			hsa_circ_0016164	chr1:204538256-204538280[+]	
			hsa_circ_0016165	chr1:204538256-204538280[+]	
hsa-miR-195-5p	NM_001759	CCND2	hsa_circ_0025055	chr12:4301754-4301780[+]	10
			hsa_circ_0025057	chr12:4301754-4301780[+]	
			hsa_circ_0025059	chr12:4301754-4301780[+]	
	NM_001145306	CDK6	hsa_circ_0081030	chr7:92608750-92608770[-]	6
	NM_001259	CDK6	hsa_circ_0081031	chr7:92608750-92608770[-]	6
	NM_001145306	CDK6	hsa_circ_0081030	chr7:92609660-92609682[-]	2
	NM_001259	CDK6	hsa_circ_0081031	chr7:92609660-92609682[-]	2
	NM_001114122	CHEK1	hsa_circ_0024793	chr11:125655348-125655365[+]	3
	NM_213647	FGFR4	hsa_circ_0075147	chr5:177093218-177093238[+]	2
	NM_001243186	PIM1	hsa_circ_0076213	chr6:37174579-37174599[+]	12
	NM_002648	PIM1	hsa_circ_0076214	chr6:37174579-37174599[+]	12
			hsa_circ_0076215	chr6:37174579-37174599[+]	
			hsa_circ_0076216	chr6:37174579-37174599[+]	
hsa-miR-424-5p	NM_001759	CCND2	hsa_circ_0025055	chr12:4301759-4301780[+]	10

			hsa_circ_0025057	chr12:4301759-4301780[+]	
			hsa_circ_0025059	chr12:4301759-4301780[+]	
	NM_001145306	CDK6	hsa_circ_0081030	chr7:92608750-92608771[-]	6
	NM_001259	CDK6	hsa_circ_0081031	chr7:92608750-92608771[-]	6
	NM_001145306	CDK6	hsa_circ_0081030	chr7:92609660-92609681[-]	2
	NM_001259	CDK6	hsa_circ_0081031	chr7:92609660-92609681[-]	2
	NM_001114121	CHEK1	hsa_circ_0024791	chr11:125655346-125655365[+]	3
			hsa_circ_0024794	chr11:125655346-125655365[+]	
	NM_001114122	CHEK1	hsa_circ_0024793	chr11:125655346-125655365[+]	3
	NM_213647	FGFR4	hsa_circ_0075147	chr5:177093217-177093238[+]	2
	NM_001243186	PIM1	hsa_circ_0076213	chr6:37174578-37174599[+]	12
	NM_002648	PIM1	hsa_circ_0076214	chr6:37174578-37174599[+]	12
			hsa_circ_0076215	chr6:37174578-37174599[+]	
			hsa_circ_0076216	chr6:37174578-37174599[+]	
hsa-miR-497-5p	NM_001759	CCND2	hsa_circ_0025055	chr12:4301760-4301780[+]	9
			hsa_circ_0025057	chr12:4301760-4301780[+]	
			hsa_circ_0025059	chr12:4301760-4301780[+]	
	NM_001145306	CDK6	hsa_circ_0081030	chr7:92608750-92608773[-]	6
	NM_001259	CDK6	hsa_circ_0081031	chr7:92608750-92608773[-]	6
	NM_001145306	CDK6	hsa_circ_0081030	chr7:92609660-92609679[-]	2
	NM_001259	CDK6	hsa_circ_0081031	chr7:92609660-92609679[-]	2

	NM_001114121	CHEK1	hsa_circ_0024791	chr11:125655345-125655365[+]	3
			hsa_circ_0024794	chr11:125655345-125655365[+]	
	NM_001114122	CHEK1	hsa_circ_0024793	chr11:125655345-125655365[+]	3
	NM_213647	FGFR4	hsa_circ_0075147	chr5:177093217-177093238[+]	2
	NM_001243186	PIM1	hsa_circ_0076213	chr6:37174579-37174599[+]	12
	NM_002648	PIM1	hsa_circ_0076214	chr6:37174579-37174599[+]	12
			hsa_circ_0076215	chr6:37174579-37174599[+]	
			hsa_circ_0076216	chr6:37174579-37174599[+]	
hsa-miR-6838-5p	NM_001759	CCND2	hsa_circ_0025055	chr12:4301759-4301780[+]	10
			hsa_circ_0025057	chr12:4301759-4301780[+]	
			hsa_circ_0025059	chr12:4301759-4301780[+]	
	NM_001145306	CDK6	hsa_circ_0081030	chr7:92608750-92608770[-]	6
	NM_001259	CDK6	hsa_circ_0081031	chr7:92608750-92608770[-]	6
	NM_001145306	CDK6	hsa_circ_0081030	chr7:92609660-92609681[-]	2
	NM_001259	CDK6	hsa_circ_0081031	chr7:92609660-92609681[-]	2
	NM_001114121	CHEK1	hsa_circ_0024791	chr11:125655344-125655365[+]	4
	NM_001114122	CHEK1	hsa_circ_0024794	chr11:125655344-125655365[+]	4
			hsa_circ_0024793	chr11:125655344-125655365[+]	
	NM_213647	FGFR4	hsa_circ_0075147	chr5:177093216-177093238[+]	2
	NM_002648	PIM1	hsa_circ_0076213	chr6:37174578-37174599[+]	12
	NM_001243186	PIM1	hsa_circ_0076214	chr6:37174578-37174599[+]	12

			hsa_circ_0076215	chr6:37174578-37174599[+]	
			hsa_circ_0076216	chr6:37174578-37174599[+]	
hsa-miR-19b-3p	NM_001759	CCND2	hsa_circ_0025055	chr12:4302312-4302332[+]	15
			hsa_circ_0025057	chr12:4302312-4302332[+]	
			hsa_circ_0025059	chr12:4302312-4302332[+]	
	NM_001759	CCND2	hsa_circ_0025055	chr12:4301471-4301493[+]	10
			hsa_circ_0025057	chr12:4301471-4301493[+]	
			hsa_circ_0025059	chr12:4301471-4301493[+]	
	NM_001114122	CHEK1	hsa_circ_0024793	chr11:125657004-125657028[+]	21
hsa-miR-193b-3p	NM_001145306	CDK6	hsa_circ_0081030	chr7:92608951-92608973[-]	5
	NM_001259	CDK6	hsa_circ_0081031	chr7:92608951-92608973[-]	5
	NM_001114122	CHEK1	hsa_circ_0024793	chr11:125655821-125655842[+]	2
hsa-miR-34a-5p	NM_001145306	CDK6	hsa_circ_0081030	chr7:92614045-92614068[-]	13
	NM_001259	CDK6	hsa_circ_0081031	chr7:92614045-92614068[-]	13
	NM_001145306	CDK6	hsa_circ_0081030	chr7:92605952-92605977[-]	1
	NM_001259	CDK6	hsa_circ_0081031	chr7:92605952-92605977[-]	1
	NM_002393	MDM4	hsa_circ_0016156	chr1:204538253-204538274[+]	7
			hsa_circ_0016159	chr1:204538253-204538274[+]	
			hsa_circ_0016160	chr1:204538253-204538274[+]	
			hsa_circ_0016162	chr1:204538253-204538274[+]	
			hsa_circ_0016164	chr1:204538253-204538274[+]	

			hsa_circ_0016165	chr1:204538253-204538274[+]	
	NM_002393	MDM4	hsa_circ_0016156	chr1:204553921-204553942[+]	6
			hsa_circ_0016160	chr1:204553921-204553942[+]	
			hsa_circ_0016162	chr1:204553921-204553942[+]	
			hsa_circ_0016164	chr1:204553921-204553942[+]	
			hsa_circ_0016165	chr1:204553921-204553942[+]	
			hsa_circ_0016166	chr1:204553921-204553942[+]	
			hsa_circ_0016167	chr1:204553921-204553942[+]	
	NM_002393	MDM4	hsa_circ_0016156	chr1:204553228-204553249[+]	5
			hsa_circ_0016160	chr1:204553228-204553249[+]	
			hsa_circ_0016162	chr1:204553228-204553249[+]	
			hsa_circ_0016164	chr1:204553228-204553249[+]	
			hsa_circ_0016165	chr1:204553228-204553249[+]	
			hsa_circ_0016166	chr1:204553228-204553249[+]	
			hsa_circ_0016167	chr1:204553228-204553249[+]	
	NM_002393	MDM4	hsa_circ_0016156	chr1:204553954-204553976[+]	2
			hsa_circ_0016160	chr1:204553954-204553976[+]	
			hsa_circ_0016162	chr1:204553954-204553976[+]	
			hsa_circ_0016164	chr1:204553954-204553976[+]	
			hsa_circ_0016165	chr1:204553954-204553976[+]	
			hsa_circ_0016166	chr1:204553954-204553976[+]	

			hsa_circ_0016167	chr1:204553954-204553976[+]	
hsa-miR-320a	NM_001759	CCND2	hsa_circ_0025055	chr12:4302921-4302944[+]	1
			hsa_circ_0025057	chr12:4302921-4302944[+]	
			hsa_circ_0025059	chr12:4302921-4302944[+]	
	NM_004456	EZH2	hsa_circ_0004930	chr7:148849348-148849367[-]	1
hsa-miR-423-5p	NM_002393	MDM4	hsa_circ_0016156	chr1:204550070-204550091[+]	2
			hsa_circ_0016160	chr1:204550070-204550091[+]	
			hsa_circ_0016162	chr1:204550070-204550091[+]	
			hsa_circ_0016164	chr1:204550070-204550091[+]	
			hsa_circ_0016165	chr1:204550070-204550091[+]	
			hsa_circ_0016166	chr1:204550070-204550091[+]	
			hsa_circ_0016167	chr1:204550070-204550091[+]	
hsa-miR-98-5p	NM_002393	MDM4	hsa_circ_0016156	chr1:204557032-204557053[+]	18
			hsa_circ_0016160	chr1:204557032-204557053[+]	
			hsa_circ_0016162	chr1:204557032-204557053[+]	
			hsa_circ_0016164	chr1:204557032-204557053[+]	
			hsa_circ_0016165	chr1:204557032-204557053[+]	
			hsa_circ_0016166	chr1:204557032-204557053[+]	
			hsa_circ_0016167	chr1:204557032-204557053[+]	
	NM_002393	MDM4	hsa_circ_0016156	chr1:204538258-204538280[+]	7
			hsa_circ_0016159	chr1:204538258-204538280[+]	

			hsa_circ_0016160	chr1:204538258-204538280[+]	
			hsa_circ_0016162	chr1:204538258-204538280[+]	
			hsa_circ_0016164	chr1:204538258-204538280[+]	
			hsa_circ_0016165	chr1:204538258-204538280[+]	
hsa-let-7b-5p	NM_002393	MDM4	hsa_circ_0016156	chr1:204557032-204557053[+]	18
			hsa_circ_0016160	chr1:204557032-204557053[+]	
			hsa_circ_0016162	chr1:204557032-204557053[+]	
			hsa_circ_0016164	chr1:204557032-204557053[+]	
			hsa_circ_0016165	chr1:204557032-204557053[+]	
			hsa_circ_0016166	chr1:204557032-204557053[+]	
			hsa_circ_0016167	chr1:204557032-204557053[+]	
	NM_002393	MDM4	hsa_circ_0016156	chr1:204538260-204538280[+]	7
			hsa_circ_0016159	chr1:204538260-204538280[+]	
			hsa_circ_0016160	chr1:204538260-204538280[+]	
			hsa_circ_0016162	chr1:204538260-204538280[+]	
			hsa_circ_0016164	chr1:204538260-204538280[+]	
			hsa_circ_0016165	chr1:204538260-204538280[+]	
hsa-miR-185-5p	NM_001759	CCND2	hsa_circ_0025055	chr12:4304925-4304946[+]	4
			hsa_circ_0025057	chr12:4304925-4304946[+]	
			hsa_circ_0025059	chr12:4304925-4304946[+]	
	NM_001759	CCND2	hsa_circ_0025055	chr12:4304082-4304104[+]	2

			hsa_circ_0025057	chr12:4304082-4304104[+]	
			hsa_circ_0025059	chr12:4304082-4304104[+]	
	NM_001080416	MYBL1	hsa_circ_0084640	chr8:66563280-66563303[-]	1
hsa-miR-145-5p	NM_001145306	CDK6	hsa_circ_0081030	chr7:92605261-92605283[-]	9
	NM_001259	CDK6	hsa_circ_0081031	chr7:92605261-92605283[-]	9
hsa-miR-214-3p	NM_001145306	CDK6	hsa_circ_0081030	chr7:92608752-92608774[-]	6
	NM_001259	CDK6	hsa_circ_0081031	chr7:92608752-92608774[-]	6
	NM_213647	FGFR4	hsa_circ_0075147	chr5:177093213-177093236[+]	2
	NM_001243186	PIM1	hsa_circ_0076213	chr6:37174577-37174597[+]	12
	NM_002648	PIM1	hsa_circ_0076214	chr6:37174577-37174597[+]	12
			hsa_circ_0076215	chr6:37174577-37174597[+]	
			hsa_circ_0076216	chr6:37174577-37174597[+]	
	NM_001243186	PIM1	hsa_circ_0076213	chr6:37175312-37175334[+]	2
	NM_002648	PIM1	hsa_circ_0076214	chr6:37175312-37175334[+]	2
			hsa_circ_0076215	chr6:37175312-37175334[+]	
			hsa_circ_0076216	chr6:37175312-37175334[+]	
	NM_001243186	PIM1	hsa_circ_0076213	chr6:37173065-37173085[+]	1
	NM_002648	PIM1	hsa_circ_0076214	chr6:37173065-37173085[+]	1
			hsa_circ_0076215	chr6:37173065-37173085[+]	
			hsa_circ_0076216	chr6:37173065-37173085[+]	
hsa-miR-155-5p	NM_001080416	MYBL1	hsa_circ_0084640	chr8:66562582-66562604[-]	1

hsa-miR-24-3p	NM_017556	FBLIM1	hsa_circ_0010089	chr1:15784743-15784764[+]	3
			hsa_circ_0010090	chr1:15784743-15784764[+]	
			hsa_circ_0010091	chr1:15784743-15784764[+]	
			hsa_circ_0010092	chr1:15784743-15784764[+]	
			hsa_circ_0010099	chr1:15784743-15784764[+]	
			hsa_circ_0010101	chr1:15784743-15784764[+]	
			hsa_circ_0010102	chr1:15784743-15784764[+]	
			hsa_circ_0010103	chr1:15784743-15784764[+]	
	NM_017556	FBLIM1	hsa_circ_0010089	chr1:15786486-15786508[+]	2
			hsa_circ_0010090	chr1:15786486-15786508[+]	
			hsa_circ_0010091	chr1:15786486-15786508[+]	
			hsa_circ_0010092	chr1:15786486-15786508[+]	
			hsa_circ_0010099	chr1:15786486-15786508[+]	
			hsa_circ_0010101	chr1:15786486-15786508[+]	
			hsa_circ_0010102	chr1:15786486-15786508[+]	
			hsa_circ_0010103	chr1:15786486-15786508[+]	
hsa-miR-9-5p	NM_002393	MDM4	hsa_circ_0016156	chr1:204556170-204556192[+]	1
			hsa_circ_0016160	chr1:204556170-204556192[+]	
			hsa_circ_0016162	chr1:204556170-204556192[+]	
			hsa_circ_0016164	chr1:204556170-204556192[+]	
			hsa_circ_0016165	chr1:204556170-204556192[+]	

		hsa_circ_0016166	chr1:204556170-204556192[+]
		hsa_circ_0016167	chr1:204556170-204556192[+]
