# Supplementary material for: In Silico Identification of circPIM1/miR-16-5p/miR-195-5p/PIM1 Feed-Forward Loop in Recurrent Grade 2 Meningioma
Source: Int J Mol Sci. 2025 Aug 26;26(17):8263. doi: 10.3390/ijms26178263 (PMC12428460; doi:10.3390/ijms26178263)
Supplement: Supplementary file 1 [file ijms-26-08263-s001.zip › Table S5-200825_Rev01_corrected.pdf]

Table S5. Known dysregulation of candidate circRNAs in cancer.

circRNA isoform (circBase ID)	CircRNA host gene name	Involvement in disease[1]	PMID	circRNA dysregulation
hsa_circ_0010090	FBLIM1	hepatocellular carcinoma (HCC)	32907351; 30053867	Up-regulated in (i) exosomes isolated from the serum of HCC patients compared with healthy volunteers; (ii) HCC tissues compared with matched adjacent normal tissues (iii) HepG2, 7402, 97H, SNU-387 and Huh7 HCC cell lines compared with normal hepatocyte cell lines LO2 and THLE-2
hsa_circ_0044516	COL1A1	prostate cancer (PCa)	31625175; 34993722	Up-regulated in (i) PCa tissues compared with normal tissues; (ii) PCa cell lines DU145 and LNCaP compared with normal prostate epithelial cell line RWPE-1; (iii) exosomes isolated from the blood of PCa patients compared with healthy volunteers; (iv) exosomes isolated from culture medium of PCa cell lines PC3, 22RV1, DU145, 2B4, C4-2 compared with exosomes isolated from culture medium of normal prostate epithelial cell line RWPE-1
hsa_circ_0044516	COL1A1	gastric cancer (GC)	33140323; 33417162	Up-regulated in (i) GC tissues compared with adjacent normal tissues; (ii) MGC-803, MNK-45, SGC-7901, HGC-27, AGS and NCI-N87 GC cell lines compared with immortalized normal gastric mucosa cell line GES-1
hsa_circ_0044516	COL1A1	lung cancer	34258296	Up-regulated in (i) lung cancer tissues compared with adjacent normal tissues; (ii) A549, SPCA1, H1299, H460, and H23 lung cancer cell lines compared with normal lung bronchial epithelial cell line BEAS-2B

hsa_circ_0044520 and hsa_circ_0044529	COL1A1	laryngeal squamous cell carcinoma (LSCC)	30282067; 35037694	Up-regulated in (i) LSCC tissue compared with normal laryngeal mucosa tissues from patients with laryngeal trauma (ii) LSCC cell lines SNU899 and TU177 compared with human normal oral epithelial keratinocytes cell line HOK

[1] From circRNADisease v2.0 database (accessed August, 2024)
