# Supplementary material for: In Silico Identification of circPIM1/miR-16-5p/miR-195-5p/PIM1 Feed-Forward Loop in Recurrent Grade 2 Meningioma
Source: Int J Mol Sci. 2025 Aug 26;26(17):8263. doi: 10.3390/ijms26178263 (PMC12428460; doi:10.3390/ijms26178263)
Supplement: Supplementary file 1 [file ijms-26-08263-s001.zip › Table S6_Rev01.pdf]

**Table S6.** Biological function of the 11 candidate circRNA host genes belonging to the 34HR-MNG biomarker and their dysregulation in MNG and other cancers.

| Host-gene | Biological Function | Dysregulated expression within the 34HR-MNG biomarker | Dysregulated expression in MNG or other tumors | PMID |
|---|---|---|---|---|
| CCND2 | Checkpoint mediated cell cycle arrest (G1/S transition) in response to DNA damage or the presence of unreplicated DNA. Involved in cell cycle, cell proliferation. It interacts with and is involved in the phosphorylation of tumor suppressor protein Rb | Suppressed | Upregulation | 11485924, 35936751 |
| CDK6 | Positive regulator of cell cycle progression (G1 phase progression and G1/S transition) | Suppressed | Upregulation | 25148008 |
| CHEK1 | Checkpoint mediated cell cycle arrest in response to DNA damage or the presence of unreplicated DNA | Enriched | Upregulation | 36115056 |
| CKS2 | Positive cell cycle control. It binds to the catalytic subunit of the cyclin dependent kinases and is essential for their biological function | Enriched | Upregulation | 29590631, 22964784, 35712492, 26894859 |
| COL1A1 | It is involved in the formation of collagen fibers | Enriched | Upregulation | 23285163, 27475668 |
| EZH2 | Member of the Polycomb-group (PcG) family. It is involved in the maintenance of the transcriptional repressive state of genes over successive cell generations. It also associates with the embryonic ectoderm development protein, playing a role in CNS physiological development | Enriched | Upregulation | 32729292, 36029259, 28195122, 33970242 |
| FBLIM1 | It is involved in the assembly and stabilization of actin filaments and likely plays a role in modulating cell adhesion, cell morphology and cell motility | Enriched | Upregulation | *38152333 |
| FGFR4 | Acts as a cell-surface receptor for fibroblast growth factors and plays a role in the regulation of cell proliferation, differentiation and migration, and in the regulation of lipid metabolism, bile acid biosynthesis, glucose uptake, vitamin D metabolism and phosphate homeostasis, and cell | Enriched | Upregulation | 35213084 |

| | | | | |
|---|---|---|---|---|
| | migration, influencing mitogenesis and differentiation | | | |
| MDM4 | Binds the p53 tumor suppressor protein and inhibits its activity, by binding its transcriptional activation domain and has been shown to be overexpressed in a variety of human cancers | Enriched | Upregulation | 17473381 |
| MYBL1 | It is involved in positive regulation of transcription by RNA polymerase II | Enriched | Upregulation | *23633565 |
| PIM1 | It belongs to the Ser/Thr protein kinase family. It is involved in cell survival and cell proliferation thus providing a selective advantage in tumorigenesis | Enriched | Upregulation | 27158663 |

*The literature does not report articles highlighting the involvement of this gene in MNG. PMIDs are referred to involvement in other CNS tumors such as gliomas.
