# Supplementary material for: In Silico Identification of circPIM1/miR-16-5p/miR-195-5p/PIM1 Feed-Forward Loop in Recurrent Grade 2 Meningioma
Source: Int J Mol Sci. 2025 Aug 26;26(17):8263. doi: 10.3390/ijms26178263 (PMC12428460; doi:10.3390/ijms26178263)
Supplement: Supplementary file 1 [file ijms-26-08263-s001.zip › Table S7_Rev01.pdf]

Table S7. Number of MR-miRNA binding sites retrieved within candidate MR-circRNAs. The median number of binding sites for a same and different candidate MR-miRNA is highlighted in bold within the table.

Candidate MR-circRNA (circBase ID)	Host gene	Total number of binding sites for the same or different candidate MR-miRNA
hsa_circ_0081030	CDK6	26
hsa_circ_0081031	CDK6	26
hsa_circ_0025055	CCND2	20
hsa_circ_0025057	CCND2	20
hsa_circ_0025059	CCND2	20
hsa_circ_0076213	PIM1	13
hsa_circ_0076214	PIM1	13
hsa_circ_0076215	PIM1	13
hsa_circ_0076216	PIM1	13
hsa_circ_0016156	MDM4	12
hsa_circ_0016160	MDM4	12
hsa_circ_0016162	MDM4	12
hsa_circ_0016164	MDM4	12
hsa_circ_0016165	MDM4	12
hsa_circ_0044510	COL1A1	11
hsa_circ_0044511	COL1A1	11
hsa_circ_0044512	COL1A1	11
hsa_circ_0024793	CHEK1	10
hsa_circ_0044509	COL1A1	10
hsa_circ_0044518	COL1A1	9
hsa_circ_0044519	COL1A1	9
hsa_circ_0075147	FGFR4	8
hsa_circ_0044520	COL1A1	8
hsa_circ_0044522	COL1A1	8
hsa_circ_0044524	COL1A1	8
hsa_circ_0016166	MDM4	8
hsa_circ_0016167	MDM4	8
hsa_circ_0044504	COL1A1	7
hsa_circ_0044505	COL1A1	7
hsa_circ_0044506	COL1A1	7
hsa_circ_0044507	COL1A1	7
hsa_circ_0044508	COL1A1	7
hsa_circ_0044526	COL1A1	7
hsa_circ_0044527	COL1A1	7

hsa_circ_0044528	COL1A1	7
hsa_circ_0044529	COL1A1	7
hsa_circ_0044535	COL1A1	7
hsa_circ_0044536	COL1A1	7
hsa_circ_0044539	COL1A1	7
hsa_circ_0024791	CHEK1	6
hsa_circ_0024794	CHEK1	6
hsa_circ_0044517	COL1A1	6
hsa_circ_0044534	COL1A1	6
hsa_circ_0044543	COL1A1	6
hsa_circ_0044548	COL1A1	6
hsa_circ_0044549	COL1A1	6
hsa_circ_0044514	COL1A1	**5**
hsa_circ_0044515	COL1A1	**5**
hsa_circ_0044516	COL1A1	**5**
hsa_circ_0044533	COL1A1	**5**
hsa_circ_0044547	COL1A1	**5**
hsa_circ_0010089	FBLIM1	4
hsa_circ_0010090	FBLIM1	4
hsa_circ_0010091	FBLIM1	4
hsa_circ_0010092	FBLIM1	4
hsa_circ_0010099	FBLIM1	4
hsa_circ_0010101	FBLIM1	4
hsa_circ_0010102	FBLIM1	4
hsa_circ_0010103	FBLIM1	4
hsa_circ_0084640	MYBL1	4
hsa_circ_0044521	COL1A1	4
hsa_circ_0044523	COL1A1	4
hsa_circ_0044525	COL1A1	4
hsa_circ_0044551	COL1A1	4
hsa_circ_0044552	COL1A1	4
hsa_circ_0044553	COL1A1	4
hsa_circ_0044554	COL1A1	4
hsa_circ_0044555	COL1A1	4
hsa_circ_0044557	COL1A1	4
hsa_circ_0044558	COL1A1	4
hsa_circ_0044559	COL1A1	4
hsa_circ_0044560	COL1A1	4
hsa_circ_0044561	COL1A1	4
hsa_circ_0016159	MDM4	4
hsa_circ_0044530	COL1A1	3

hsa_circ_0044531	COL1A1	3
hsa_circ_0044532	COL1A1	3
hsa_circ_0044537	COL1A1	3
hsa_circ_0044538	COL1A1	3
hsa_circ_0044503	COL1A1	3
hsa_circ_0087441	CKS2	2
hsa_circ_0087442	CKS2	2
hsa_circ_0044540	COL1A1	2
hsa_circ_0044541	COL1A1	2
hsa_circ_0044542	COL1A1	2
hsa_circ_0044544	COL1A1	2
hsa_circ_0044545	COL1A1	2
hsa_circ_0044546	COL1A1	2
hsa_circ_0044502	COL1A1	2
hsa_circ_0025054	CCND2	1
hsa_circ_0025056	CCND2	1
hsa_circ_0025058	CCND2	1
hsa_circ_0044562	COL1A1	1
hsa_circ_0044563	COL1A1	1
hsa_circ_0044513	COL1A1	1
hsa_circ_0004930	EZH2	1
