# Supplementary material for: In Silico Identification of circPIM1/miR-16-5p/miR-195-5p/PIM1 Feed-Forward Loop in Recurrent Grade 2 Meningioma
Source: Int J Mol Sci. 2025 Aug 26;26(17):8263. doi: 10.3390/ijms26178263 (PMC12428460; doi:10.3390/ijms26178263)
Supplement: Supplementary file 1 [file ijms-26-08263-s001.zip › Table S8_Rev01.pdf]

**Table S8.** Number of candidate MR-circRNAs biogenerated by MR-host genes.

| **Host gene of candidate MR-circRNA** | **Number of candidate MR-circRNAs biogenerated by MR-host genes** |
|---|---|
| COL1A1 | 60 |
| MDM4 | 8 |
| FBLIM1 | 8 |
| CCND2 | 6 |
| PIM1 | 4 |
| CHEK1 | 3 |
| CDK6 | 2 |
| CKS2 | 2 |
| FGFR4 | 1 |
| MYBL1 | 1 |
| EZH2 | 1 |
