# Supplementary material for: In Silico Identification of circPIM1/miR-16-5p/miR-195-5p/PIM1 Feed-Forward Loop in Recurrent Grade 2 Meningioma
Source: Int J Mol Sci. 2025 Aug 26;26(17):8263. doi: 10.3390/ijms26178263 (PMC12428460; doi:10.3390/ijms26178263)
Supplement: Supplementary file 1 [file ijms-26-08263-s001.zip › Table S9-200825_Rev01_corrected.pdf]

Table S9. List of primer sequences used in this study (Fw: forward; Rev: reverse).

Transcript Name	Fw sequence (5’-3’)	Rev sequence (5’-3’)	Source of primer sequences
PIM1	GAGAAGGACCGGATTTCCGAC	CAGTCCAGGAGCCTAATGACG	Designed through Primer-BLAST (https://www.ncbi.nlm.nih.gov/tools/primer-blast/)
hsa_circ_0076215	CTGTGTTTTGAGCAGCAGGT	AGTCCAGGAGCCTAATGACG	Designed through circPrimer 2.0 (https://www.bio-inf.cn/)
hsa_circ_0076216	CTGTGTTTTGAGCAGCAGGT	CCACACCCTTTCCTCCCTAG	Designed through circPrimer 2.0 (https://www.bio-inf.cn/)
GAPDH	GTCAGCCGCATCTTCTTTTG	GCGCCCAATACGACCAAATC	Taken from ref. [16]
