# Supplementary figures and images for: In Silico Identification of circPIM1/miR-16-5p/miR-195-5p/PIM1 Feed-Forward Loop in Recurrent Grade 2 Meningioma
Source: Int J Mol Sci. 2025 Aug 26;26(17):8263. doi: 10.3390/ijms26178263 (PMC12428460; doi:10.3390/ijms26178263)

**Figure S3.** Dot plot visualization of Pim1 based upon the GSE150219 dataset [58].

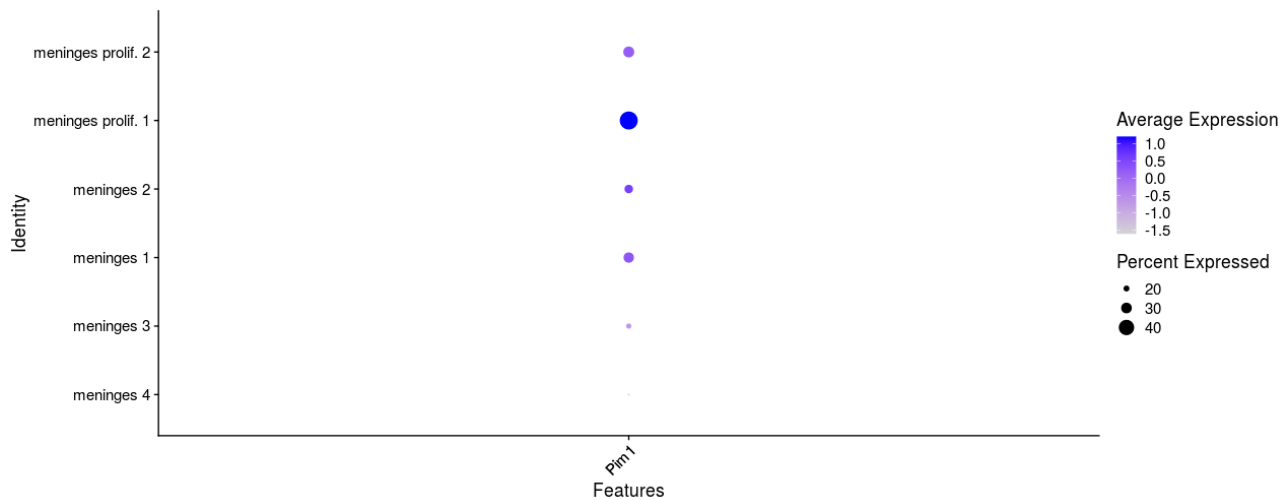

Supplement: Supplementary file 1 [file ijms-26-08263-s001.zip › Figure S3_Rev02.pdf]
